# Obtaining and managing data sets for individual participant data meta-analysis: scoping review and practical guide

**DOI:** 10.1186/s12874-020-00964-6

**Published:** 2020-05-12

**Authors:** Matthew Ventresca, Holger J. Schünemann, Fergus Macbeth, Mike Clarke, Lehana Thabane, Gareth Griffiths, Simon Noble, David Garcia, Maura Marcucci, Alfonso Iorio, Qi Zhou, Mark Crowther, Elie A. Akl, Gary H. Lyman, Viktoria Gloy, Marcello DiNisio, Matthias Briel

**Affiliations:** 1 Department of Health Research Methods, Evidence, and Impact, McMaster University, Hamilton, Ontario Canada; 2 Centre for Trials Research, School of Medicine, Cardiff University, Cardiff, Wales, UK; 3 Northern Ireland Hub for Trials Methodology Research and Cochrane Individual Participant Data Meta-analysis Methods Group, Queen’s University Belfast, Belfast, UK; 4 Wales Cancer Trials Unit, School of Medicine, Cardiff University, Wales, UK; Faculty of Medicine, University of Southampton, Southampton General Hospital, Southampton, UK; 5 Marie Curie Palliative Care Research Centre, Cardiff University, Cardiff, Wales, UK; 6 Department of Medicine, University of Washington School of Medicine, Seattle, WA USA; 7 Department of Medicine, McMaster University, Hamilton, Ontario Canada; 8 Department of Internal Medicine, American University of Beirut, Beirut, Lebanon; 9 Department of Medicine, University of Washington School of Medicine, Seattle, Washington, USA; 10 Hutchinson Institute for Cancer Outcomes Research, Fred Hutchinson Cancer Research Center, Seattle, Washington USA; 11 Basel Institute for Clinical Epidemiology and Biostatistics, Department of Clinical Research, University of Basel and University Hospital Basel, Basel, Switzerland; 12 Department of Medicine and Ageing Sciences, University G. D’Annunzio, Chieti-Pescara, Italy

**Keywords:** Individual participant data meta-analysis, Data collection, Data sharing, Systematic review, Practical guide

## Abstract

**Background:**

Shifts in data sharing policy have increased researchers’ access to individual participant data (IPD) from clinical studies. Simultaneously the number of IPD meta-analyses (IPDMAs) is increasing. However, rates of data retrieval have not improved. Our goal was to describe the challenges of retrieving IPD for an IPDMA and provide practical guidance on obtaining and managing datasets based on a review of the literature and practical examples and observations.

**Methods:**

We systematically searched MEDLINE, Embase, and the Cochrane Library, until January 2019, to identify publications focused on strategies to obtain IPD. In addition, we searched pharmaceutical websites and contacted industry organizations for supplemental information pertaining to recent advances in industry policy and practice. Finally, we documented setbacks and solutions encountered while completing a comprehensive IPDMA and drew on previous experiences related to seeking and using IPD.

**Results:**

Our scoping review identified 16 articles directly relevant for the conduct of IPDMAs. We present short descriptions of these articles alongside overviews of IPD sharing policies and procedures of pharmaceutical companies which display certification of Principles for Responsible Clinical Trial Data Sharing via Pharmaceutical Research and Manufacturers of America or European Federation of Pharmaceutical Industries and Associations websites. Advances in data sharing policy and practice affected the way in which data is requested, obtained, stored and analyzed.

For our IPDMA it took 6.5 years to collect and analyze relevant IPD and navigate additional administrative barriers. Delays in obtaining data were largely due to challenges in communication with study sponsors, frequent changes in data sharing policies of study sponsors, and the requirement for a diverse skillset related to research, administrative, statistical and legal issues.

**Conclusions:**

Knowledge of current data sharing practices and platforms as well as anticipation of necessary tasks and potential obstacles may reduce time and resources required for obtaining and managing data for an IPDMA. Sufficient project funding and timeline flexibility are pre-requisites for successful collection and analysis of IPD. IPDMA researchers must acknowledge the additional and unexpected responsibility they are placing on corresponding study authors or data sharing administrators and should offer assistance in readying data for sharing.

## Background

A meta-analysis aims to combine findings from different studies to obtain a more precise estimate of the average effect of an intervention or the size of an association, or to explore how and why results differ across studies [1]. There are several ways of synthesizing study data [2, 3]. Generally, a meta-analysis may combine study level data or individual participant level data. Study level meta-analyses combine estimates from multiple studies to generate a summary estimate. Individual participant data (IPD) meta-analyses (MA) combine data from each specific participant from multiple studies into a single dataset for further analysis [4]. IPDMA are considered the “gold standard” [5–9] and possibly preferred to study level meta-analyses because they allow researchers to use the most current and comprehensive data, verify the findings of previous investigations, apply uniform definitions and analyses across studies, and avoid potential ecological bias when investigating interactions between interventions and patient-level characteristics (effect modifications, subgroup effects) [7, 8, 10–12]. Similar to systematic reviews and study level meta-analyses, IPDMAs often influence practice guidelines and the design of new trials [13, 14].

Ideally, an IPDMA should be based on IPD from all studies included in a systematic review, regardless of the study designs chosen for the systematic review [15]. An IPDMA can be conducted on data from randomized trials, observational studies, including registries, and other study designs although there are risks and challenges in combining these different study designs. However, fewer than half of systematic reviews with IPDMA, published between 1987 and 2015, retrieved data from at least 80% of relevant studies and from at least 80% of relevant participants [16]. The number of IPDMAs increased over this period [17], but data retrieval rates remained unchanged [16, 18]. Inability to include eligible studies compromises the systematic review’s purpose, decreases study power, and leads to healthcare decisions based on an incomplete, potentially biased data sample (studies with available data may differ from those whose data are not available) [10, 19, 20]. However, analysis combining individual and study level data may mitigate these effects [3, 21].

Since the first IPDMA guide published in 1995 [7], researchers have found that the process of obtaining, managing, and organizing IPD is typically the most resource intensive and time consuming step and may require years to complete [1, 4, 7, 16, 22]. Thus, many systematic reviews rely on study level data even though sharing IPD and conducting IPDMA would be more useful [8, 20, 23–31].

Study participants have also understood the benefits of data sharing and are generally willing for this to happen, but may fear the loss of data confidentiality, misuse, or sharing without consent [32–35]. Governments [36, 37], research organizations [38–40], scientific journals [38, 41–46] and the pharmaceutical industry [47, 48] have developed data sharing policies. The Institute Of Medicine (IOM) has released four recommendations to guide responsible data sharing [49]: (1) maximize the benefits of clinical trials while minimizing the risks of sharing clinical trial data, (2) respect individual participants whose data are shared, (3) increase public trust in clinical trials and the sharing of trial data, and (4) conduct the sharing of clinical trial data in a fair manner. In July 2013, amid some criticism [50, 51], the European Federation of Pharmaceutical Industries and Associations (EFPIA) and the Pharmaceutical Research and Manufacturers of America (PhRMA) issued a joint statement describing the principles of responsible data sharing [47]. Several pharmaceutical companies and academic institutions are now working to handle data sharing requests in a more timely, better organized, and increasingly transparent manner by using the services of independent data sharing platforms or creating their own [52–76].

Based on a review of the literature and our own experience with conducting IPDMAs, our goal was to provide practical guidance for researchers to successfully obtain IPD of eligible studies and to reduce resources required for IPDMA. We describe the key challenges and propose solutions to navigate obstacles commonly associated with IPDMA in the light of recent changes in data sharing policy and practice [16, 47, 77].

## Methods

### Search strategy and inclusion criteria

After delays during data acquisition for our recent IPDMA of the use heparin in patients with cancer [77], we noticed changes in data sharing policy and practice [47, 78] in clinical trial data access and began to log our setbacks and solutions. We then conducted systematic searches of MEDLINE, Embase, and the Cochrane Library (from inception of each database until January 2019) to identify publications describing strategies to obtain IPD or IPDMA best practice. An experienced research librarian helped design a comprehensive search strategy using MeSH terms and text words (Additional file 1) without any language restrictions.

Eligibility criteria included (1) articles describing IPDMA best practice including topics such as planning, cost, required time, common burdensome tasks, or administrative issues; (2) systematic reviews describing trends in IPDMA including topics such as IPD retrieval rates; (3) quantitative or qualitative studies describing strategies, barriers, or facilitators to obtain IPD from industry or investigator-sponsored studies; and (4) case reports describing authors’ attempts to obtain IPD. We excluded IPDMAs reporting on a specific clinical question or statistical papers, e.g. studies describing different techniques of combining IPD with study level data.

### Screening

Two methodologically trained reviewers (MV and VG) independently screened titles and abstracts. If eligibility was suspected or unclear, we obtained full texts. Three reviewers (MV, MB, VG) screened full texts independently and in duplicate. Disagreements were resolved by discussion and consensus. From included articles we extracted information providing practical guidance for researchers to successfully obtain IPD and to make the conduct of IPDMA more efficient. Our scoping review adheres to the Preferred Reporting Items for Systematic reviews and Meta-Analyses extension for Scoping Reviews (PRISMA-ScR) guidelines [79].

### Additional sources

Several publications examining specific data sharing issues outside of the context of an IPDMA (e.g. data sharing models or author reimbursement in general) did not meet the inclusion criteria for the scoping review but were referenced to provide additional context. In addition, we searched websites of pharmaceutical companies which have publicly certified with PhRMA or EFPIA as having complied with the Principles for Responsible Data Sharing [47], data repositories [52, 76, 80], and industry organizations [81, 82] for press releases and other information about policies for sharing IPD. Finally, we drew from the authors’ experiences in providing, seeking, or using IPD - in particular, a recently conducted IPDMA investigating heparin use among cancer patients [77]. Based on the systematically identified literature, policy websites, and our own experience we developed practical guidance for IPDMA researchers that we structured according to the course of tasks when conducting an IPDMA.

**Fig. 1:**
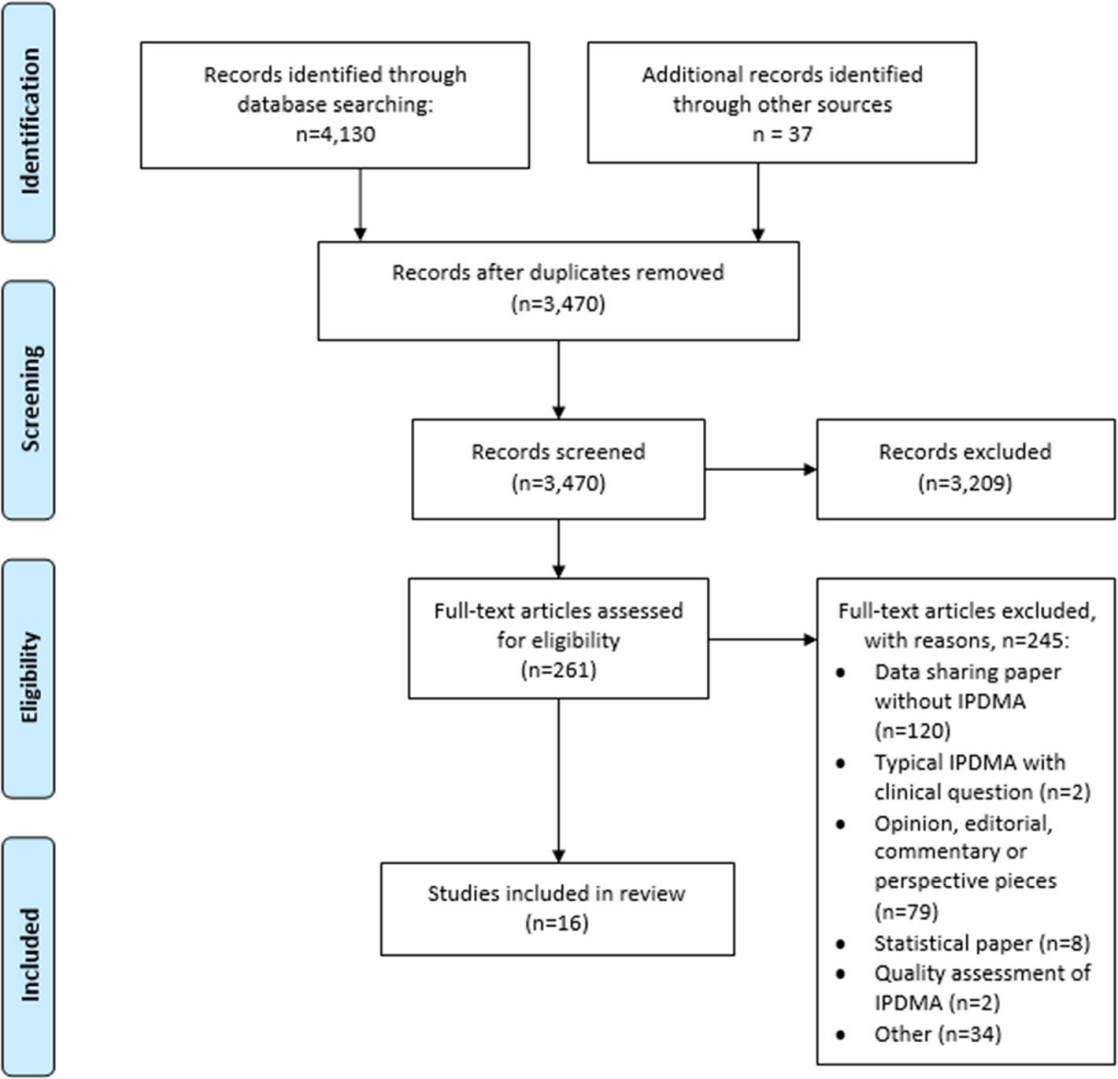
PRISMA flow diagram

**Table 1:** Included articles with direct relevance to guide researchers in the conduct of IPDMA

Study	Description
Abo-Zaid et al. (2012). “Individual participant data meta-analysis of prognostic factor studies: state of the art?” [22]	Systematic review of IPDMAs of prognostic factors aimed at describing the conduct, evaluation and commonly experienced challenges.
Berlin et al. (2014). “Bumps and bridges on the road to responsible sharing of clinical trial data.” [128]	Literature review providing guidance on the process of obtaining and combining datasets from different sources.
Clarke (2005). “Individual patient data meta-analyses.” [1]	Systematic review describing the rationale of IPDMA and processes for obtaining IPD.
Higgins JPT and Green S. Cochrane Handbook for Systematic Reviews of Interventions Version 5.1.0 [updated March 2011]; Available from: www.handbook.cochrane.org . [cited 2018 January 11]. [15]	The Cochrane Handbook provides guidance to authors performing Cochrane Intervention reviews. Chapter 18 describes IPDMAs, including the collaboration process.
Huang, et al. (2014). “Distribution and epidemiological characteristics of published individual patient data meta-analyses.” [18]	Survey of published IPDMAs until August 2012 describing their distribution and epidemiologic characteristics.
Jaspers and Degraeuwe (2014). “A failed attempt to conduct an individual patient data meta-analysis.” [23]	Case report describing the process of pursuing data and lessons learned from an IPDMA, which could not be completed.
Nevitt et al. (2017). “Exploring changes over time and characteristics associated with data retrieval across individual participant data meta-analyses: Systematic review.” [16]	Systematic review of IPDMAs conducted until August 2015, which identifies study factors significantly associated with obtaining a high proportion of IPD.
Polanin (2018). “Efforts to retrieve individual participant data sets for use in a meta-analysis result in moderate data sharing but many data sets remain missing.” [129]	Meta-analysis of IPDMAs, which examines the success rate of obtaining IPD solely through direct contact with study authors.
Polanin and Terzian (2019). “A data-sharing agreement helps to increase researchers’ willingness to share primary data: results from a randomized controlled trial.” [130]	Randomized controlled trial assessing the effect of IPDMA authors providing a data sharing agreement on primary author data sharing.
Polanin and Williams (2016). “Overcoming obstacles in obtaining individual participant data for meta-analysis.” [131]	Review, that provides solutions to barriers encountered while obtaining IPD for IPDMA.
Riley et al. (2010). “Meta-analysis of individual participant data: rationale, conduct, and reporting.” [4]	Description of rationale, conduct and reporting standards of IPDMA, which also describes recent trends in published IPDMA.
Ross (2016). “Clinical research data sharing: what an open science world means for researchers involved in evidence synthesis.” [26]	Commentary on general data sharing trends and predictions, including some barriers to identifying, obtaining and combining datasets for IPDMA.
Stewart and Clarke (1995). “Practical methodology of meta-analyses (overviews) using updated individual patient data. Cochrane Working Group.” [7]	The first practical guide describing IPDMA conduct, including discussion of planning, obtaining and analyzing IPD.
Tierney et al. (2015). “Individual Participant Data (IPD) Meta-analyses of Randomised Controlled Trials: Guidance on Their Use.” [132]	An updated guide describing IPDMA conduct, including discussion of planning, obtaining and analyzing IPD.
Veroniki et al. (2016). “Contacting authors to retrieve individual patient data: Study protocol for a randomized controlled trial.” [133]	Study protocol for a randomized controlled trial comparing data acquisition techniques.
Young and Hopewell (2011). “Methods for obtaining unpublished data.” [134]	Review of studies, that examines techniques for obtaining IPD by contacting primary study authors.

**Table 2:** Summary recommendations for obtaining individual participant data

**Requesting data through personal contact or data sharing repository**	
Review the data sharing policy of the study’s sponsor organization.	
Data sharing requests can be submitted using a professional email account or through a data sharing repository.	
Contact data repositories to inquire about datasets not listed for request.	
In addition to the IPD, consider requesting access for the study protocol, analysis plans, analysis-ready dataset, meta-data, annotated case report forms, and clinical study report.	
Multiple contact attempts occurring over months or years may be required. Send emails on behalf of well-known researchers, those with personal connections to study authors, or from well-known research organizations to assist in garnering a response.	
Discuss data sharing through teleconferences or in-person meetings rather than fragmented email correspondence whenever possible.	
Offer to complete the essential data sharing tasks and provide necessary funding for researchers who may lack the time or organizational resources to share data.	
Record the names, affiliations, contact information and roles of internal and external data sharing stakeholders throughout the data sharing process.	
**Incentives for data contributors**	
Offer authorship or other incentives (eg. financial, acknowledgement) to those deserving credit for generating primary data.	
**Setting up a data sharing agreement**	
Adapt previous data sharing agreements or existing templates to suit specific studies and institutional policies of study sponsors. Seek assistance form your institution’s industry liaison office.	
**Time to data retrieval and refused requests**	
Continue to contact study stakeholders until a refusal to share data has been confirmed.	
Seek reasoning for denied data sharing requests and attempt to develop solutions to data sharing barriers.	
Effective communication and negotiation with primary study stakeholders may allow sharing of IPD before or immediately after publication of primary study results.	
Document non-responses and refused data sharing requests for report in results publications.	
**Managing retrieved IPD**	
Review the primary study protocol, results publications, clinical study reports, annotated case report forms and other shared files before and alongside data processing.	
Datasets which could not be shared may be incorporated into analysis using methods which combine study level and IPD.	
Allow data sharing organizations to review and comment on analysis prior to publication, ensuring accurate interpretation of shared data.	
Identify projects emerging from IPDMA before results publication or prior to deletion of shared study data.	
**Confidentiality and data storage**	
Research local laws and sponsor policies pertaining to the storage and sharing of personally identifying information.	

## Results

The systematic search of our scoping review yielded 3470 titles and abstracts (Fig. 1). We identified 16 eligible articles that are presented in Table 1 together with a short description. In Table 2 we summarize our main recommendations for researchers when retrieving data sets for IPDMAs and provide corresponding explanations and elaborations in the following sections.

### Identifying relevant studies

A sensitive search for all eligible studies, published and unpublished, is crucial for all systematic reviews to minimize publication bias [135]. Cochrane provides useful techniques to identify and obtain published as well as unpublished study data [15, 134]. Trial registries or regulatory bodies may be instrumental in identifying unpublished eligible studies and constitute an initial contact point (e.g. corresponding author or data sharing administrator) for data sharing requests. See Additional file 1 for detailed information about the International Clinical Trials Registry Platform and Additional file 1 on the United States Food and Drug Administration and the European Medicines Association. In principle, there are two approaches to obtain IPD: (1) direct contact with study authors, or (2) requests via a data repository [131].

The data collection process of our own IPDMA occurred between October 2012 and June 2016 [77]. All data requests were placed by contacting study authors except for two of the 19 studies, which we learned by reviewing each organization’s data sharing policies, required use of the online data request portal clinicalstudydatarequest.com (CSDR). For all studies, we requested access to the clinical trial data, meta-data, study protocol, annotated case report forms, and clinical study report.

### Requesting study data through personal contact

Analysis of data sharing requests submitted solely through study authors indicates that 58% of requests are successful [129]. Qualitative research examining useful techniques to obtain unpublished data indicates that concise, friendly requests which minimize additional responsibilities (e.g. drafting a data sharing agreement, converting old datasets to digital format) for the primary study author and attempt to establish a personal connection would be more likely to receive a response [136]. IPDMA authors typically attempt contact several times before quitting; the most persistent tried every 6 months for 2 to 3 years [130, 131, 136, 137]. From our own experience, obtaining data sets through personal contact required as little as 4 months and as much as 4 years. Every corresponding author or study sponsor responded to our request; but we made repeated contact attempts via email, fax or phone. In some cases, we reviewed the institution’s data sharing request policy to identify additional data sharing contacts (eg. organizational email address such as datasharing@Amgen.com) or alternative request procedures (eg. submitting a request through an independent data repository such as clinicalstudydatarequest.com). A description of our approach to correspondence and a sample email request are available in Table 3 and Additional file 1, respectively. Email correspondence is often fragmented and delayed. Organizing phone or in-person meetings, e.g. at conferences, was often useful when explaining the IPDMA’s purpose and anticipated tasks to study authors before any data was shared and whenever detailed discussions of complicated issues (e.g. security of data storage servers) was necessary. These conversations also led to the development of personal relationships with study authors which we felt eased correspondence throughout the data sharing and analysis process.

**Table 3:** Approach to email correspondence

Suggestion	
Corresponding authors are typically the first point of contact when requesting study data via email	
Send emails using a professional organizational email account rather than a personal email account (eg. @gmail.com, @hotmail.com)	
When possible, send emails on behalf of a well-known research organization, from someone with professional authority or from a personal acquaintance	
Include the primary investigator, research coordinator and key team members in requesting emails	
Include obvious keywords in the subject line allowing easy message retrieval	
Clearly define a purpose and exclude use of acronyms as well as emotional cues	
Express concern for alternative duties and avoid rude, irritating, or unprofessional language	
Describe recognition for data sharing	
Request a teleconference or in-person meeting to discuss several issues in a brief period	
Attach a study protocol and other important documents to requesting emails	
For each study, generate a list of contacts and corresponding responsibilities	

Primary authors may lack time, funding, or organizational resources to support essential data sharing tasks (e.g. transferring data to an electronic format, drafting data sharing agreement). Our IPDMA research team offered assistance with these tasks whenever possible. Recording contact information and roles of data sharing stakeholders (e.g. administrators, statisticians, industry liaisons, ethical and legal representatives) is essential. This eased subsequent communication which often occurred years after the first data request as the IPDMA progressed to publication.

### Requesting study data via data repository or data sharing administrator

In our IPDMA, two datasets were requested and approved through CSDR, a consortium of clinical study sponsors and funders which facilitates responsible data sharing [138]. IPDMA authors may be required to directly contact a data repository or data sharing administrator and submit a full study proposal rather than make a simple inquiry [139]. Initially, we reviewed the list of studies with data available to be requested but neither dataset was available from the study’s sponsor. For one study, the sponsor had not yet properly curated the data. Despite this, we contacted CSDR via email, followed by a teleconference, and this process was expedited at our request. For the second, the study sponsor was in the process of establishing a presence on CSDR and shared data after doing so.

In our experience, the process of submitting data requests on CSDR takes approximately 30 to 60 min; it was intuitive, and directions were available [78, 140]. Our request package identified the specific study by the title and National Clinical Trial number and included our study protocol, timeline, funding sources, description of research team members’ experience and roles, conflicts of interest, and publication plans. Knowledge of jurisdictional laws (e.g. Personal Information Protection and Electronic Documents Act and General Data Protection Regulation) and collaboration with legal representatives was required before submitting data sharing requests and while negotiating data sharing agreements. Approximately 4 months were needed to process each data sharing request and finalize the data sharing agreement, consistent with CSDR estimates [120, 141]. After finalizing the data sharing agreement, our questions pertaining to data sharing processes or system technical difficulties were typically responded to within 1 day.

As of December 31, 2019, 1429 requests were made for data on CSDR which were not listed by the study’s sponsors; 559 submissions were approved and 843 denied, while 51 are still under consideration [142]. Of companies which have received at least 40 requests for non-listed studies, the reported lowest percentage of approval is 9%, (Eisai), and the highest 74% (GlaxoSmithKline) [142]. Geifman et al. reported the data request process via CSDR to be unnecessarily lengthy, while requests submitted through Project Data Sphere, an alternative data sharing platform devoted to cancer related clinical trials [76], required only days before data access was provided [143].

The joint PhRMA and EFPIA statement represents the minimum clinical transparency standard, but participation is voluntary [47, 144, 145]. Industry sponsors which are members of PhRMA or EFPIA are more likely to publicize a data sharing policy and make trial data eligible for sharing [146, 147]. For pharmaceutical companies publicly certifying compliance with the Principles for Responsible Clinical Trial Data Sharing through the PhRMA or EFPIA websites [83, 84], the data access points, summary of data made available, and date from which the pharmaceutical company’s IPD sharing policy applies is exhibited in Table 1 and Table 2. Certified pharmaceutical companies with data procedures that could not be confirmed through additional internet searching are not included. Each sponsor’s specific policy should be referred to for a complete review of available data. A sponsor’s exclusion from Table 4 or Table 5 is not meant to indicate they are not wholly committed to data sharing, but that as of March 5, 2019, certification of their compliance with the Principles for Responsible Clinical Trial Data Sharing was not confirmed through PhRMA or EFPIA websites [83, 84]. Repositories may also provide access to study data which is sponsored, generated or stored by governments, universities, charities and research organizations [52, 80].

**Table 4:** Data availability of pharmaceutical companies displaying certification via PhRMA or EFPIA websites which solicit data requests via online data sharing platform [47, 83, 84]

Pharmaceutical company	Clinical data made available	Publicized date of earliest available data
**Request point:**https://clinicalstudydatarequest.com [52]		
Astellas [55, 85]	Phase 1, 2, 3 and 4 studies for indications which have been approved by the US and, or EU	January 1, 2010
Bayer [56]	All trials required for regulatory approval	January 1, 2014
Chugai [86]	All sponsored clinical trials	January 1, 2014
Eisai [61]	Phase 2, 3 and 4 studies required for regulatory approval which have been approved by the US and, or EU.	January 1, 2014
GlaxoSmithKline [64, 87, 88]	All global interventional studies All interventional studies since 2013 Other studies where data are provided to researchers	December 1, 2000
Novartis [89]	Phase 2 and 3 studies required for regulatory approval in the EU or US Requested studies must support the indication	January 1, 2014
Roche [75]	All phase 2 and 3 studies or phase 4 studies required for regulatory approval. Products terminated from development.	January 1, 1999
Sanofi [67]	All trials, for approved indications, required for regulatory approval in the US and EU	January 1, 2014
Shionogi {Shionogi & Co. Ltd., 2018 #3717}	Phase 1, 2, 3, and 4 studies used for regulatory approval in the US, EU, and Japan	February 1, 2019
Sumitomo Dainippon Pharma Co, Ltd. [90]	Phase 2, 3, and 4 interventional clinical studies included in the submission package for approved medications in the US, EU, or Japan	January 1, 2014
UCB [70]	Phase 2, 3, and 4 study data for approved medicines and indications	November 1, 2008
Viiv Healthcare [65]	Phase 2, 3, and 4 study data for approved medications	November 1, 2017
Research funders^a^	Phase 1, 2, 3, and 4 interventional clinical studies Phase 1, 2, 3, and 4 interventional clinical studies for terminated compounds	January 1, 2010
**Request point:** https://yoda.yale.edu/how-request-data [91]		
Johnson & Johnson [72, 92, 93]	Phase 2, 3 and 4 studies for products approved in the US and EU	January 1, 1990^b^
**Request point:**https://vivli.org^c^ [80]		
Abbvie [53]	Phase 2, 3 and 4 interventional clinical studies for medicinal products and indications which received authorization in US or EU	May 1, 2004
Biogen [94]	Phase 1, 2, 3 and 4 interventional clinical trials for products and indications submitted to and approved in the US and EU.	January 1, 2004
Boehringer Ingelheim [58]	All trials with published results	January 1, 1998
Celgene [60, 95]	Studies supporting indications approved in the US and EU	January 1, 2014
Daiichi-Sankyo [96]	Phase 2, 3 and 4 interventional clinical studies submitted for approved medications in US, EU or Japan	January 1, 2014
GlaxoSmithKline [64, 88]	Global interventional studies Interventional studies evaluating medicines, starting in or after 2013 Consumer healthcare studies completed on or after January 1, 2018	December 1, 2000
Johnson & Johnson [72, 92, 93]	Phase 2, 3 and 4 studies for products approved in the US and EU	January 1, 1990^b^
Lilly [63]	Phase 2, 3 and 4 studies submit for regulatory approval to FDA on or after 1999 Phase 2, 3, 4 global studies after January 2007 Phase 2, 3, 4 regional studies for drugs approved in US and EU since January 1, 2014	January 1, 1999
Pfizer [66, 97]	Global interventional studies conducted for medicines, vaccines, and medical devices which were terminated or are approved in the US or EU	September 1, 2007
Takeda [70]	Phase 1,2,3 and 4 trials which support products approved in the US, EU, and/or Japan and products terminated from development	January 1, 2005
UCB [70]	Phase 2, 3, and 4 study data for medicines and indications approved in the US and EU	January 1, 2007
**Request point:**https://astrazenecagroup-dt.pharmacm.com/DT/Home/Login		
AstraZeneca [98–100]	Phase 1, 2, 3, or 4 studies for approved indications in the US, EU, or Japan	January 1, 2009*
**Request point**: https://biogen-dt-external.pharmacm.com/DT/Home		
Biogen [57]	Phase 1, 2, 3, or 4 studies for discontinued compounds or those approved in the US and EU	January 1, 2014
**Request point:**https://fasttrack.force.bms.com/		
Bristol-Myers Squibb [59, 101]	Phase 1, 2, 3, or 4 study data for medicines and indications approved in the US or EU	January 1, 2008
**Request point**: https://www.celgeneclinicaldatasharing.com		
Celgene [60, 95]	Study data for compounds and indications approved in the US and EU	January 1, 2014
**Request point:**http://www.chiesi.com/en/chiesi-clinical-trial-data-request-portal/		
Chiesi [102]	Study data for medications approved by the FDA or EMA	January 1, 2015
**Request point:**https://www.emdgroup.com/en/research/our-approach-to-research-and-development/healthcare/clinical-trials/commitment-responsible-data-sharing.html		
EMD Serono [63, 103]	Study data for products and indications approved in the US and EU	January 1, 2014
**Request point:**https://clinicaltrials.menarini.com/en-US/Home/Register		
Menarini [104, 105]	Study data for medications and indications approved in the US and EU	Unclear
**Request point:**http://engagezone.msd.com/		
Merck & Co. [73]	Study data submit for regulatory approval in the US and EU for approved indications	September 1, 2007
**Request point:**https://www.purduepharma.com/healthcare-professionals/clinical-trials/#request-trial-data		
Purdue Pharmaceuticals [74]	Phase 2, 3 or 4 study data for drug products and their approved uses in the US for approved indications	January 1, 2014
**Request point:** https://errs.regeneron.com/external		
Regeneron [106]	Approved medicines and indications with publicly disclosed results	Unclear
**Request point:**https://clinicaltrials.servier.com/data-request-portal/login/		
Servier [107]	Study data for approved medications or indications in European Economic Area or US	January 1, 2014

^a^Funders include the Bill and Melinda Gates Foundation, Cancer Research UK, Medical Research Council, and Wellcome Trust

^b^Electronic data is available as far back as 1990. Study data prior to 1990 are only available in paper format and are not readily accessible

^c^Vivli.org also provides access to data sponsored, stored or generated by BioLINCC, Critical Path Institute, Cure Duchenne, Doris Duke Charitable Foundation, Duke University, Harvard University, ImmPort, Johns Hopkins University, Project Data Sphere, the Leona M. and Harry B. Helmsley Charitable Trust and the University of California San Francisco

**Table 5:** Pharmaceutical companies displaying certification via PhRMA or EFPIA websites, which solicit data requests through email [47, 83, 84]

Pharmaceutical company and access point	Advertised trial data made available	Publicized date of earliest available data
Almirall [108] R&D@almirall.com	Study data for approved medications and indications in the US or EU	January 1, 2014
Amgen [54, 109] datasharing@Amgen.com	Study data submit for regulatory approval in the US and EU for approved indications	Unclear
Bial [110] clinical.trials@bial.com	Study data for approved medications and indications in the US or EU	Unclear
Grunenthal [111] clinicaltrialportal@grunenthal.com	Study data submit in support of licensed treatments in the US or EU	July 15, 2014
Leo Pharma [112, 113] disclosure@leo-pharma.com	Study data for any approved product or products from discontinued trials which began since 2014	January 1, 2000
Lundbeck [114] clinicaldataaccess@lundbeck.com	Study data for approved medications and indications in the US or EU	January 1, 2014
Novo Nordisk [115, 116] irb-secretariat@novonordisk.com	Study data for product indications approved in the US and EU	January 1, 2001
Orion Pharma [117] Unclear request point	Study data for medications which have obtained received market authorisation	Unclear
Otsuka [118] DT-inquiry@otsuka.jp	Study data submit to the US or EU for regulatory approval	January 1, 2014
Shire [68] clinicaltrialdata@shire.com	Study data for compounds and indications approved in the US and EU	January 1, 2014
Vifor pharma [119] Unclear request point	Provide academic researchers access to clinical trial data upon request	Unclear

Examining the data sharing procedures of certified pharmaceutical companies, 26 use at least one internal or external online portal to manage data sharing requests, including clinicalstudydatarequest.com (12), vivli.org (11), yoda.yale.edu (1), fasttrack-bms.force.com (1), https://biogen-dt-external.pharmacm.com/DT/Home (1) and https://www.purduepharma.com/healthcare-professionals/clinical-trials/#request-trial-data (1). Data requests for the remainder of certified pharmaceutical companies are solicited via email. In Table 3 we describe the data request review processes from each pharmaceutical company certified through PhRMA or EFPIA. As of January, 31, 2020, 3123 studies were available on request through CSDR [142]. Vivli, an independent non-profit data-sharing and analytics platform, lists over 4900 studies [148]. Pharmaceutical companies with data procedures that could not be confirmed through internet searching are not included in Table 6.

**Table 6:** Data request review process of pharmaceutical companies displaying certification via PhRMA or EFPIA websites which solicit data requests via online data sharing platform [47, 83, 84]

Request point/pharma company	Review process	
Clinical Study Data Request affiliates^a^ [120, 121]	Research Proposals are checked and reviewed in 3 stages: **Stage 1:** by the Wellcome Trust which is the secretariat for the Independent Review Panel (IRP) **Stage 2**: by the study Sponsors/Funders **Stage 3**: by the IRP.	
Vivli affiliates [219]^b^	All data requests proceed through three steps during the data request review. **Step 1:** Vivli Administrator form check — Ensure all required fields of the data request form are completed. **Step 2:** Data Contributor Review — Check feasibility of fulfilling request. **Step 3:** Approving Entity, Scientific Panel, or Independent Review Panel — Reviews based upon the merits of the research proposal	
Abbvie [53]	All requests from qualified researchers for access to AbbVie clinical data and information will be managed by Vivli and AbbVie. In cases where we reject a particular request based on scientific merit, the request, along with the record of our denial of the request, shall be forwarded to the Access to Clinical Research Information Board (ATCRIB) for a final decision, according to the ATCRIB charter. The ATCRIB is composed of scientists and/or health care professionals who are not AbbVie employees.	
Almirall [108]	All requests will be evaluated independently on a case-by-case.	
Amgen [54, 109]	Research proposals will be reviewed by a committee of internal advisors. For clinical trials that are subject to agreements with co-development partners, Amgen will liaise with the applicable partners regarding any data sharing requests. In general, Amgen does not support external research questions that involve access to individual patient level data for the purpose of re-evaluating safety and efficacy issues already addressed in the product labelling. If the outcome of the internal review is to decline the request, a Data Sharing Independent Review Panel will arbitrate and make the final decision.	
AstraZeneca [100]	An independent Scientific Review Board to review and approve requests. The Scientific Review Board will review requests that go back as far as 2009 through this process. All other requests for data beyond that will continue to be reviewed by AstraZeneca on a case-by-case basis.	
Bial [110]	Each request will be evaluated by an independent Scientific Review Board and will be based on criteria that balance the need for scientific development with the need to protect patient privacy.	
Biogen [57, 122]	Biogen reviews all data requests internally based on the criteria set forth in our Clinical Trial Transparency and Data Sharing Policy. Requests that are denied in whole or in part are then sent to an independent external review body, whose decision will be made transparent.	
Bristol-Myers Squibb [59, 123]	The request/proposal is currently being reviewed internally by a qualified panel of Bristol-Myers Squibb experts. If the proposal is considered within scope, the request will undergo an additional review by the independent review committee.	
Celgene [60, 95]	A group of individuals selected by the Celgene Clinical Trial Data Sharing Steering Committee composed of external experts to provide an unbiased review of research proposals submitted by researchers to ensure that the proposals are robust, scientifically sound with a valid and clearly defined hypothesis and include both an analysis and publication plan.	
Chiesi [102]	An appointed Chiesi Evaluation Committee starts the assessment of the research proposal. In case of a negative evaluation, but no direct competition is envisaged, Chiesi forwards the assessment to a Scientific Review Board, composed by qualified researchers who are not Chiesi employees.	
EMD Serono [63, 124]	Researchers’ requests will be evaluated initially by an internal committee at EMD Serono, which may decide to approve the request. If the EMD Serono committee denies the request, the request will be escalated to the EMD Serono Scientific Review Board for a second review (de novo). The Board shall include scientists and/or healthcare professionals who are not employees of EMD Serono.	
Grunenthal [125]	Requests for access to clinical data will be subject to assessment and approval by a Grünenthal Board and then by an independent Scientific Review Board.	
Janssen [126]	During the Review, the YODA Project will evaluate submitted requests and associated registration materials to ensure that all required information has been provided. All requests for data will undergo review upon receipt by the YODA Project. During this review, the YODA Project will evaluate submitted requests and associated registration materials to ensure that all required information has been provided and that the Research Proposal has scientific merit. Requests will undergo External Review if the YODA Project is unable to verify the scientific merit of the Research Proposal.	
Leo Pharma [112, 113]	The evaluation of the data request and the decision on access to data is made by the external Patient and Scientific Review Board. The Patient and Scientific Review Board comprise three highly experienced scientists while two seats are allocated to representatives of patient associations. The decision by the Patient and Scientific Review Board is made independently of LEO Pharma.	
Lundbeck [114]	An external scientific review board is responsible for assessing and granting requests from qualified scientific and medical researchers. If the scientific review board rejects a request, the scientific review board can advise a resubmission.	
Menarini Group [105]	All requests will be reviewed internally by a qualified panel of Menarini Group experts (Scientific Secretariat) and then passed to an Independent Review Committee (IRC) of external experts for further review.	
Merck & Co. [73]	Completed applications will be reviewed by MSD with Input as needed from an External Scientific Review Board comprised of non-MSD scientists or physicians.	
Novo Nordisk [115, 116]	The Independent Review Board assesses all complete requests and approves or rejects the proposal without any interference from Novo Nordisk.	
Orion Pharma [117]	After a marketing authorisation has been granted to our new drug, we allow access to our patient-level data based on a scientific review of the request and the proposal from the external research group consisting of qualified scientific and medical researchers.	
Otsuka [118]	Research proposals requesting patient-level data are reviewed by an Independent Review Panel at Western Institutional Review Board Copernicus Group. Research proposals for non-listed studies are examined on a case-by-case basis by Otsuka in consultation with the Independent Review Panel.	
Pfizer [66, 97]	An internal Pfizer Review Committee conducts the initial review of in scope requests. Any request approved by Pfizer will not require a secondary review by the Independent Review Panel. Pfizer is piloting the use of an Independent Review Panel during 2014. The Panel will review any proposal declined, or partially approved, by Pfizer. The role of the Panel is to review the application, the rationale for Pfizer’s response, and to make a final decision. The decision of the Panel will be binding.	
Purdue Pharma [74]	The Purdue Scientific Review Board (SRB) will adjudicate all requests for Information. The SRB will consist of Purdue employees selected by the Chief Medical Officer (CMO) from relevant departments, such as Research and Development, Medical Affairs, Law, and Ethics & Compliance, and two researchers or external experts who are not employees of Purdue.	
Regeneron [106, 127]	**The Regeneron Investigator-Initiated Study Program, which is comprised of a cross functional team, will evaluate data sharing requests on a case-by-case basis.**	Unclear
Servier [107]	Servier will conduct the initial review, including scientific qualification of the researcher, the robustness and scientific merit of the research proposal, the ability of the requested data to answer the research question, and the technical feasibility. If Servier partially approves or declines the request, we send our decision to the IRB for review. The decision made by the IRB is final and binding for Servier.	
Shire [68]	Once Shire assesses the validity of the researcher’s data request and determines appropriate consent(s) exists for requested product(s) and indication(s), an internal team made of subject matter experts will review the eligibility of the proposed research against the criteria below and render a decision. In cases where the validity of the researcher or proposed request is in question, Shire will defer the request to an external Independent Review Panel for a final, objective opinion.	
Vifor pharma [119]	Unable to locate.	

^a^Industry affiliates include Astellas, Bayer, Chugai, Eisai, GlaxoSmithKline, Novartis, Roche, Sanofi, Shionogi, Sumitomo Dainippon, Takeda, UCB, and Viiv healthcare. Non-industry affiliates include Bill and Melinda Gates Foundation, Cancer Research UK, Medical Research Council, and Wellcome Trust

^b^Industry affiliates include Abbvie, Biogen, Boehringer Ingelheim, Celgene, Daiichi-Sankyo, GlaxoSmithKline, Johnson & Johnson, Lilly, Pfizer, Takeda, Tempus, UCB. Non-industry affiliates include BioLINCC, Critical Path Institute, Cure Duchenne, Doris Duke Foundation, Duke University, Harvard University, ImmPort, Johns Hopkins University, Project Data Sphere, The Leona M. and Harry B. Helmsley Charitable Trust and University of California San Francisco

### Incentives for data contributors

Study authors and data curators who generated, managed and shared data, and provided commentary on findings make considerable efforts that should be recognized. Given the role in data collection and interpretation of data, we offered authorship or acknowledgement on relevant publications to corresponding authors and individuals the corresponding author deemed worthy of authorship or acknowledgement. Researchers generally agree that trialists who share data deserve recognition and propose several methods including, direct financial payments, publication incentives, and consideration of previous data sharing practices by funding agencies, and consideration by academic institutions in decisions regarding career promotions, or the possibility of penalties to large organizations, such as fines or suspension of a product’s market authorization, for those refusing data sharing [27, 136, 149–156]. Authorship also enables primary researchers to contribute to the manuscript before publication and reduces anxiety about a lack of control over data and fellow researchers’ ability to understand shared data or IPDMA results [153, 154].

There are several administrative, standardization, human resources and opportunity costs to properly preserve a data repository, manage requests and prepare data for additional analysis which IPDMA authors may be asked to contribute to [157–161]. Academic researchers are expected to pay between $30,000 and $50,000 annually to list up to 20 studies on CSDR [162]. Vivli asks researchers and pharmaceutical companies to pay between $2000 and $4500 per listed study [163]. We obtained funding to offer reimbursement of minor expenses associated with data sharing (e.g. shipping fees for datasets which corresponding authors preferred not to send electronically) but did not offer direct payment for time required to prepare study data, negotiate data sharing agreements, or respond to analytical questions. Funding for these tasks was also not requested by any of the collaborating parties. Offering a small financial incentive, 100 Canadian Dollars, to primary study authors has not improved IPD retrieval rates [137].

### Setting up a data sharing agreement

Data sharing agreements describe the conditions which the IPDMA research team should respect in exchange for permission to analyze specified data from a trialist or study sponsor, and are recommended when sharing data [49, 164, 165]. Data sharing agreements describe the study rationale, analysis plan, contents being exchanged, participant confidentiality, timing of data sharing, data storage and security measures, third party data sharing, intellectual property rights, publication plans and authorship, among others. We adapted previous data sharing agreements to suit the institutional policies of respective study sponsors. Eight of the 14 eligible studies utilized data sharing data sharing agreements while the remaining six did not feel it was necessary. However, we do recommend their use. We sought feedback from our institution’s industry liaison department regarding legal phrasing and implications of the data sharing agreement. Additional file 1 presents an example data sharing agreement with further details. We had to negotiate amendments to ratified agreements if institutional policies changed, if there were data sharing issues affecting agreements with others, or when we conducted additional analyses.

### Time to data retrieval and reasons for refused requests

Two of our data sharing requests were not granted (one because of ongoing analyses and the other because it could not be transferred to a shareable electronic format) and three could not be pursued because of timeline and resource restrictions. This meant that we were unable to obtain data for 18% of participants (*n* = 1763) [77]. Contacting trial authors, negotiating data sharing agreements and awaiting publication of study results are common reasons for delays. Approximately 43% of IPDMAs obtain at least 80% of IPD [16]. The IOM recommends that sponsors make available the “full data package” to external researchers no later than 18 months after trial completion and the “post-publication data package” no more than 6 months after trial completion [49]. In practice, the time until IPD become available after trial completion varies greatly. This availability is influenced by when primary results are published and when a drug’s development program is terminated or approved by regulators, among other factors [52].

Data which are commonly unavailable include commercially confidential information (information not in the public domain which may undermine the legitimate economic interests of the company [166]), and study data which were not submitted as part of a marketing authorization package [52]. Sponsors may require that secondary analysis investigate the same indication as the primary analysis because study participants have not provided consent for other investigations. Many sponsors have recognized this impediment and changed their participant consent forms accordingly [52]. Systematic reviews have identified several other technical, motivational, economic, political, legal and ethical barriers to data sharing such as inclusion of data from grey literature, increased costs due to use of commercial data sharing platforms, and advancing data anonymization standards [16, 160, 167, 168].

Authors’ motivations for accepting or rejecting data sharing requests include advancing science, improving healthcare, complying with employer, funding, or sponsor policies, participant privacy, perceived effort and personal recognition [20, 25, 49, 153, 154, 167–173]. Some argued that older trials require excessive time and resources to properly anonymize IPD, update databases to current standard or transfer data to an electronic format, assuming they have not been lost [16, 137]. Sharing of databases may be refused because datasets are too large to properly anonymize and transfer to other researchers [52, 174]. In such cases, IPDMA researchers may request only relevant variables rather than entire raw datasets which will be smaller and obstruct the ability to use multiple variables to identify a study participant.

If a request is denied, IPDMA researchers may combine IPD with study level data to examine the potential impact of studies without IPD on results and to understand the totality of the evidence [3, 19, 175–177].

### Managing retrieved IPD

Reviewing supplemental material and readying datasets is a time consuming and resource intensive task [159]. Older datasets generally require additional maintenance as they are not digitally recorded or coded to current standards. For our IPDMA, we reviewed the study protocol, publications, clinical study reports, annotated case report forms and other shared files, before and alongside data extraction to understand the dataset and ensure accuracy. Annotated case report forms are particularly helpful in understanding shared data as they connect each specific variable in a dataset to when, why, where, or how the data was collected. We logged inconsistencies and typically resolved them through discussion with study stakeholders (e.g. trial coordinators). Important inconsistencies should be described in publications following the Preferred Reporting Items for Systematic Review and Meta-Analyses of Individual Participant Data (PRISMA-IPD) statement [178].

We created a unified database that was verified by two researchers. Our data sharing agreements require that shared data will be deleted within 6 months of results publication which requires careful planning of all analyses.

In our own IPDMA, access to one study required use of the SAS Clinical Trial Data Transparency (CTDT) portal and approval from the institutional review board and trial sponsors [179, 180]. A manual is provided to assist researchers using the CTDT portal, but training is needed if researchers are unfamiliar with statistical analysis programs [180–183]. A dedicated support team is available to resolve technical issues. Analysis of data accessed through the SAS CTDT portal may require IPDMA researchers to temporarily upload remaining data to this platform. The consent of clinical trial study sponsors not using the SAS CTDT system may be required before doing so. Conversely, IPDMA researchers may also try to negotiate the download of data typically securely accessed through the CTDT system. For further review of methodology and statistical issues for IPDMA see Debray et al. 2015 [176].

### Confidentiality and data storage

In our IPDMA, we deleted information from databases that identified study participants (e.g. names or phone numbers) because storing personal information is not in the interest of study participants. Indeed, the general public and study participants worry about storing or sharing of personally identifying information, obtaining appropriate consent to use data, and relationships with the study investigators [26, 34, 184]. IPDMA researchers must be aware of local laws and sponsor policies about the storage of personally identifying information [16]. Concerns about lack of anonymity are also common when requesting data from case-studies or case-series involving fewer than 50 participants, trials of rare diseases or trials assessing genomic data [52]. Thus, all data requires storage on secure password protected servers where access is provided only to those directly involved in data analysis according to available standards [52, 185–187].

## Discussion

We conducted a scoping review of challenges and solutions to obtaining and using IPD and supplemented this with descriptions of our own experiences to guide and facilitate future IPDMA. Many of the practical issues we identified are new compared to the Cochrane IPDMA working group’s guide published by Stewart and Clarke in 1995 [7]. Technological and cultural changes have modified the ways in which researchers communicate and collaborate and the ways data are shared, managed and analyzed. Recent guidance on the use and appraisal of IPDMAs [188, 189], reporting standards [178], data sharing [49], and statistical techniques [176] have influenced these policies.

Our IPDMA identified 19 eligible studies and 10,032 eligible participants which is above the median of typical IPDMAs (i.e. 14 eligible studies and 2369 participants) [16]. Unexpected delays throughout the data gathering process resulted from challenges in communication and the need to adapt to modifications in the various sponsors’ data sharing practices, which were evolving alongside industry and government policy. Some of these changes included the joint PhRMA/EFPIA statement on the principles of responsible clinical trial data sharing [47], launch of the AllTrials campaign [190], GlaxoSmithKline introducing the first online data request platform before transitioning to CSDR in 2014 [191], and influential publications highlighting the importance of data sharing and open science [192–194].

### Limitations and strengths

This manuscript was not planned before starting the IPDMA which we use as a primary example in this work but because of the many challenges, we were encouraged to provide guidance. Thus, our solutions are based on firsthand experiences but have not been formally compared to alternatives and may not be applicable to all IPDMA. Our perspective is that of IPDMA researchers and not of trialists, sponsors, or data sharing administrators who may disagree with our proposals. Other IPDMA or study stakeholders may identify additional obstacles or solutions not described here but we have conducted a scoping review to overcome that limitation.

### Relation to other studies

We identified several publications which aimed to provide a firsthand description of specific data sharing experiences [16, 23, 143, 195–197]. For example, Savage and Vickers obtained only one of 10 requested studies and established contact with only five of 10 corresponding authors [196]. Data from the remaining four studies were not shared because preparation was too laborious, data were forbidden from being shared, or required an extensive proposal submission [196]. Jaspers and Degraeuwe described their attempt to conduct an IPDMA, which was eventually abandoned because they were able to obtain only 40% of IPD. Barriers to accessing data were similar to those we describe here and included difficulties establishing contact with study authors, denial of requests for raw datasets because of ongoing analysis or because of a lack of time and personnel to properly prepare data. Geifman et al. and Filippon et al. reported costly and repeated data sharing requests [143, 197]. Nevitt et al. performed a systematic review of IPDMAs published between 1987 and 2015, and reported that only 25% of published IPDMAs had access to all identified IPD and no improvement in data retrieval rate over time [16]. IPDMAs were associated with retrieving at least 80% of IPD if they included only randomized trials, had an authorship policy which provided an incentive to share data (e.g. co-authorship), included fewer eligible participants, and were not Cochrane Reviews.

## Conclusions

As shifts in data sharing policy and practice continue, and the number of IPDMA pursued increases, IPDMA researchers must be prepared to mitigate the effects of project delays. Knowledge of how to establish and maintain contact with study stakeholders, negotiate data sharing agreements, and manage clinical study data is required. Broader issues including designing trials for secondary analysis, participant confidentiality, data sharing models, data sharing platforms, data request review panels and recognition of primary study investigators must also be understood to ensure an IPDMA is conducted to appropriate scientific, ethical, and legal standard [128, 198–206]. We hope that a shift away from peer-to-peer requesting procedures towards data repository requests will help [207]. The discussion of specific data sharing issues such as the effectiveness of data sharing policies [208], output of data sharing endeavours [209], confidentiality of commercial information, whom data is shared with, timelines for data requests, and appropriately compensating data sharing parties must continue [26, 27, 49, 200, 210–212]. Additional research investigating the effectiveness of data acquisition techniques [133], platform features which aid the sharing of clinical trial data [213–215], incentives for data sharing [171, 208], participant broad consent for data sharing [216] and the decision to pursue an IPDMA versus study level MA is needed.

## Supplementary information

**Additional file 1.**

## Data Availability

All data generated or analysed during this study are included in this published article and its supplementary information files.

## Abbreviations

CSDR: Clinicalstudydatarequest.com
CTDT: Clinical trial data transparency
EFPI: European Federation of Pharmaceutical Industries and Associations
Embase: Excerpta Medica dataBASE
IOM: Institutes of Medicine
IPDMA: Individual participant data meta-analysis
MEDLINE: Medline Literature Analysis and Retrieval System Online
PhRMA: Pharmaceutical Research and Manufacturers of America
PRISMA-IPD: Preferred Reporting Items for a Systematic Review and Meta-Analysis of Individual Participant Data
SAS: Statistical Analysis System
US: United States

## References

[1] Clarke MJ (2005). Individual patient data meta-analyses. Best Pract Res Clin Obstet Gynaecol.

[2] Cooper HM, Hedges LV, Valentine JC (2009). The handbook of research synthesis and meta-analysis.

[3] Riley RD, Simmonds MC, Look MP (2007). Evidence synthesis combining individual patient data and aggregate data: a systematic review identified current practice and possible methods. J Clin Epidemiol.

[4] Riley RD, Lambert PC, Abo-Zaid G (2010). Meta-analysis of individual participant data: rationale, conduct, and reporting. BMJ.

[5] Simmonds MC (2005). Meta-analysis of individual patient data from randomized trials: a review of methods used in practice. Clin Trials.

[6] Tudur Smith C (2011). Individual participant data meta-analyses compared with meta-analyses based on aggregate data [abstract]. Trials.

[7] Stewart LA, Clarke MJ (1995). Practical methodology of meta-analyses (overviews) using updated individual patient data. Cochrane Working Group. Stat Med.

[8] Stewart LA, Tierney JF (2002). To IPD or not to IPD? Advantages and disadvantages of systematic reviews using individual patient data. Eval Health Prof.

[9] Tudur Smith C (2016). Individual participant data meta-analyses compared with meta-analyses based on aggregate data. Cochrane Database Syst Rev.

[10] Lyman GH, Kuderer NM (2005). The strengths and limitations of meta-analyses based on aggregate data. BMC Med Res Methodol.

[11] Mello MM (2013). Preparing for responsible sharing of clinical trial data. N Engl J Med.

[12] Stewart LA, T.J., Clarke M. Chapter 18: Reviews of individual patient data. In: Higgins JPT, Green S (editors), Cochrane Handbook for Systematic Reviews of Interventions Version 5.1.0 (updated March 2011). The Cochrane Collaboration, 2011; Available from: www.handbook.cochrane.org.

[13] Vale CL (2015). Uptake of systematic reviews and meta-analyses based on individual participant data in clinical practice guidelines: descriptive study. BMJ.

[14] Tierney JF (2015). How individual participant data meta-analyses have influenced trial design, conduct, and analysis. J Clin Epidemiol.

[15] Higgins JPT, Thomas J, Chandler J, Cumpston M, Li T, Page MJ, Welch VA (editors). Cochrane Handbook for Systematic Reviews of Interventions version 6.0 (updated July 2019). Cochrane, 2019. Available from www.training.cochrane.org/handbook. 10.1002/14651858.ED000142 PMC10284251 31643080

[16] Nevitt SJ (2017). Exploring changes over time and characteristics associated with data retrieval across individual participant data meta-analyses: systematic review. BMJ.

[17] Coady SA (2017). Use of the National Heart, Lung, and Blood Institute data repository. N Engl J Med.

[18] Huang Y (2014). Distribution and epidemiological characteristics of published individual patient data meta-analyses. PLoS One.

[19] Ahmed I, Sutton AJ, Riley RD (2012). Assessment of publication bias, selection bias, and unavailable data in meta-analyses using individual participant data: a database survey. BMJ.

[20] Rathi V (2012). Sharing of clinical trial data among trialists: a cross sectional survey. BMJ.

[21] Sutton AJ, Kendrick D, Coupland CA (2008). Meta-analysis of individual- and aggregate-level data. Stat Med.

[22] Abo-Zaid G, Sauerbrei W, Riley RD (2012). Individual participant data meta-analysis of prognostic factor studies: state of the art?. BMC Med Res Methodol.

[23] Jaspers GJ, Degraeuwe PL (2014). A failed attempt to conduct an individual patient data meta-analysis. Syst Rev.

[24] Tenopir C (2015). Changes in data sharing and data reuse practices and perceptions among scientists worldwide. PLoS One.

[25] Rathi VK (2014). Predictors of clinical trial data sharing: exploratory analysis of a cross-sectional survey. Trials.

[26] Ross JS (2016). Clinical research data sharing: what an open science world means for researchers involved in evidence synthesis. Syst Rev.

[27] Ohmann C (2017). Sharing and reuse of individual participant data from clinical trials: principles and recommendations. BMJ Open.

[28] Barnhart KT, Legro RS, Scott RT (2018). Data sharing requirements: perspectives from three authors. Fertil Steril.

[29] Sardanelli F (2018). To share or not to share? Expected pros and cons of data sharing in radiological research. Eur Radiol.

[30] Wu T (2018). Transparency and sharing individual participant data of clinical trials: a philosophical proposition about the medical study ethics and implications for clinical trials. [Chinese]. Chin J Evid Based Med.

[31] Vickers AJ (2016). Sharing raw data from clinical trials: what progress since we first asked “Whose data set is it anyway?”. Trials.

[32] Bell EA, Ohno-Machado L, Grando MA (2014). Sharing my health data: a survey of data sharing preferences of healthy individuals. AMIA Annu Symp Proc.

[33] Cheah PY (2015). Perceived benefits, harms, and views about how to share data responsibly: a qualitative study of experiences with and attitudes toward data sharing among Research staff and community representatives in Thailand. J Empir Res Hum Res Ethics.

[34] Howe N (2018). Systematic review of participants’ attitudes towards data sharing: a thematic synthesis. J Health Serv Res Policy.

[35] Mello MM, Lieou V, Goodman SN (2018). Clinical trial participants’ views of the risks and benefits of data sharing. N Engl J Med.

[36] European Medicines Agency (2014). European Medicines Agency policy on publication of clinical data for medicinal products for human use.

[37] Food and Drug Administration (FDA) (2013). Availability of Masked and De-identified Non-Summary Safety and Efficacy Data; Request for Comments.

[38] Taichman DB (2017). Data sharing statements for clinical trials: a requirement of the International Committee of Medical Journal Editors. PLoS Med.

[39] Cancer Research UK. Submission of a data sharing and preservation strategy. 2009 June 1, 2015; Available from: http://www.cancerresearchuk.org/prod_consump/groups/cr_common/@fre/@gen/documents/generalcontent/cr_016308.pdf. Accessed 15 Apr 2020.

[40] Kiley R (2017). Data sharing from clinical trials - a Research Funder's perspective. N Engl J Med.

[41] International Committee of Medical Journal (2018). Clinical Trials.

[42] Godlee F, Groves T (2012). The new BMJ policy on sharing data from drug and device trials. BMJ.

[43] PLOS Medicine. PLOS editorial and publishing policies; Available from: http://www.plosmedicine.org/static/policies.action;jsessionid=0CD27C4DE9841485620ED21B9E815D54#sharing. [cited 2014 July 1].

[44] Scientific Data. Welcome to Scientific Data; Available from: http://www.nature.com/sdata/about. [cited 2014 July 1].

[45] Loder E, Groves T (2015). The BMJ requires data sharing on request for all trials. BMJ.

[46] Fletcher J (2014). New CMAJ policy on sharing data from clinical research. CMAJ.

[47] European Federation of Pharmaceutical Industries and Associations (EFPIA) and Pharmaceutical Research and Manufacturers of America (PhRMA) (2013). Principles for resposible clinical trial data sharing: Our commitment to patients and researchers.

[48] Atzor S (2014). Clinical trial data sharing: from principles to practical implementation - an industry model. Regul Rapporteur.

[49] Lo B (2015). Sharing clinical trial data: maximizing benefits, minimizing risk. JAMA.

[50] O'Dowd A (2013). Drug industry pledge on access to trial data is met with scepticism. BMJ.

[51] Doshi P (2013). EFPIA-PhRMA's principles for clinical trial data sharing have been misunderstood. BMJ.

[52] Clinical Study Data Request. Study sponsors; Available from: https://www.clinicalstudydatarequest.com/Study-Sponsors.aspx. [cited 2014 July 4].

[53] Abbvie (2014). Clinical trials data and information sharing.

[54] Amgen (2019). Clinical Trial Transparency, Data Sharing and Disclosure Practices.

[55] Astellas (2018). About Astellas Clinical Study Results.

[56] Bayer. Clinical trial transparency policy; Available from: http://pharma.bayer.com/en/innovation-partnering/clinical-trials/transparency-policy/. [cited 2019 March 12].

[57] Biogen (2018). Biogen clinical trial transparency and data sharing policy.

[58] Boehringer-Ingelheim. Policy on transparency and publication of clinical study data: Registration of clinical studies, disclosure of results in publications and results databases and access to study data and documents; Available from: https://trials.boehringer-ingelheim.com/content/dam/internet/opu/clinicaltrial/com_EN/documents/Policy.pdf. [cited 2019 March 12].

[59] Bristol-Myers Squibb (2018). Disclosure Commitment.

[60] Celgene (2019). Clinical trials data sharing.

[61] Eisai Co. Ltd. Clinical trial disclosure; Available from: https://www.eisai.com/company/business/research/clinical/index.html. [cited 2019 March 12].

[62] Eli Lilly and Company (2014). Lilly announces increased access to clinical trials data for qualified researchers.

[63] EMD Serono (2018). Our Commitment to Responsible Clinical Trial Data Sharing.

[64] GlaxoSmithKline (2013). GSK gives update on plans to share detailed clinical trial data as part of its commitment to transparency.

[65] Viiv Healthcare (2019). We are committed to data transparency.

[66] Pfizer (2019). Data access requests.

[67] Sanofi (2019). Clinical trials and results: Our data sharing commitments.

[68] Shire (2017). Our commitment to transparency.

[69] Takeda (2015). A Tradition of Serving Patients With Integrity.

[70] UCB (2014). UCB position statement on clinical study data transparency.

[71] The Yale University Open Data Access Project (2018). Johnson & Johnson - Available Data.

[72] Janssen Global Services, L (2018). Clinical trial data transparency.

[73] Merck (2018). Procedure on access to clinical trial data.

[74] Purdue Pharma (2018). Review process for data sharing.

[75] Roche. Roche global policy on sharing of clincal study information; Available from: https://www.roche.com/dam/jcr:1c46aa73-cea0-4b9b-8eaa-e9a788ed021b/en/roche_global_policy_on_sharing_of_clinical_study_information.pdf. [cited 2019 March 13].

[76] Project Data Sphere LLC (2020). Project Data Sphere.

[77] Schunemann HJ (2016). Use of heparins in patients with cancer: individual participant data meta-analysis of randomised trials study protocol. BMJ Open.

[78] Clinical Study Data Request (2018). How to request data.

[79] Tricco AC (2018). PRISMA extension for scoping reviews (PRISMA-ScR): checklist and explanation. Ann Intern Med.

[80] Vivli (2019). Our members.

[81] European Federation of Pharmaceutical Industries and Associations (EFPIA) (2020). Home.

[82] Pharmaceutical Research and Manufacturers of America (PhRMA) (2020). PhRMA.

[83] European Federation of Pharmaceutical Industries and Associations (EFPIA) (2018). Sharing clinical trial information.

[84] Pharmaceutical Research and Manufacturers of America (PhRMA) (2017). PhRMA Principles for Responsible Clinical Trial Data Sharing – Certifications.

[85] Clinical Study Data Request (2019). Sponsor Specific Details: Astellas.

[86] Chugai Pharmaceutical Co. Ltd (2018). Clinical trial data sharing.

[87] Nisen P, Rockhold F (2013). Access to patient-level data from GlaxoSmithKline clinical trials. N Engl J Med.

[88] GlaxoSmithKline (2014). Data transparency.

[89] Novartis (2016). Novartis Position on Clinical Study Transparency – Clinical Study Registration, Results Reporting and Data Sharing.

[90] Sumitomo Dainippon Pharma (2018). Disclosure of Clinical Study Data.

[91] Yale University Open Data Access (YODA) Project. How to request data; Available from: https://yoda.yale.edu/how-request-data. [cited 2019 March 13].

[92] Johnson & Johnson (2014). Johnson & Johnson Announces Clinical Trial Data Sharing Agreement with Yale School of Medicine.

[93] Krumholz HM (2014). Give the data to the people.

[94] Biogen (2019). Biogen clinical trial transparency and data sharing policy.

[95] Celgene (2017). Clinical trial data sharing policy.

[96] Daiichi-Sankyo (2018). Clinical Trial Information Disclosure.

[97] Pfizer (2014). Clinical Trials Data Access - Independent Review Panel Charter.

[98] AstraZeneca (2018). AstraZeneca Group of Companies – Data Request Portal.

[99] Andrews N (2015). AstraZeneca Clinical Trial Transparency and External Sharing of Individual Patient-Level Data.

[100] AstraZeneca (2019). Disclosure Commitment.

[101] Pencina MJ (2016). Supporting open access to clinical trial data for researchers: the Duke Clinical Research Institute-Bristol-Myers Squibb supporting open access to researchers initiative. Am Heart J.

[102] Chiesi Group (2018). Clinical trial transparency statement.

[103] EMD Serono. Responsible data sharing instruction document; Available from: https://www.emdgroup.com/content/dam/web/corporate/non-images/business-specifics/healthcare/US/Responsible_Data_Sharing_Instruction_Document_US.pdf. [cited 2019 April 18].

[104] The Menarini Group (2018). User Registration.

[105] The Menarini Group (2018). Datasharing.

[106] Regeneron. Clinical Trial Disclosure & Data Transparency Policy Statements; Available from: https://errs.regeneron.com/external/DefaultBU1.aspx. [cited 2020 March 18].

[107] Servier (2018). Data request portal.

[108] Almirall (2019). Clinical trials.

[109] Amgen (2019). Clinical Trial Data Sharing Request.

[110] Bial (2014). Transparency.

[111] Grunenthal (2018). Clinical data sharing with researchers.

[112] Leo Pharma. LEO Pharma's Position on Public Access to Clinical Trials Information. Available from: http://www.leo-pharma.com/Home/Research-and-Development/Clinical-trial-disclosure/LEO-Pharmas-position-on-transparency.aspx. [cited 2019 March 14].

[113] Leo Pharma. How to get access to anonymised patient-level data. Available from: http://www.leo-pharma.com/Home/Research-and-Development/Clinical-trial-disclosure/Access-to-patient-level-data.aspx#Step_1:__Fasibility_assessment_by_LEO_Pharma. [cited 2019 March 14].

[114] Lundbeck (2018). Policy for scientific publications and responsible clinical trial data sharing.

[115] Novo Nordisk. How to access clinical trial datasets. Available from: https://www.novonordisk-trials.com/how-access-clinical-trial-datasets. [cited 2019 March 14].

[116] Novo Nordisk. Independent review board charter. Available from: https://www.novonordisk-trials.com/sites/novonordisk/themes/novo/img/document/Independent%20Review%20Board%20Charter.pdf. [cited 2019 March 14].

[117] Orion Corporation. Ethics in the Clinical Research Phase*.* Available from: https://www.orion.fi/en/Orion-group/Sustainability/policies/pharmaceutical-rd-ethics-policy/ethics-in-clinical-research/. [cited 2019 March 14].

[118] Otsuka Pharmaceutical Co. Ltd (2019). For researchers: Overview of researcher access to patient-level data.

[119] Vifor Pharma (2018). Responsibility highlights report 2017.

[120] Clinical Study Data Request (2018). Review of requests.

[121] Clinical Study Data Request. Independent review panel charter. Available from: https://clinicalstudydatarequest.com/Documents/Independent_Review_Panel_Charter.pdf. [cited 2018 August 1].

[122] Vivli (2019). Biogen.

[123] Duke Clinical Research Institute (2017). Bristol-Myers Squibb data sharing independent review committee (IRC) charter.

[124] EMD Serono. Summary of EMD Serono's responsible data sharing policy. [cited 2019 March 22].

[125] Grunenthal (2018). Requesting access to clinical data.

[126] Yale University Open Data Access (YODA) Project (2016). Procedures to Guide External Investigator Access to Clinical Trial Data.

[127] Regeneron (2020). Regeneron Mission Statement & Purpose of Investigator-Initiated Study Program.

[128] Berlin JA (2014). Bumps and bridges on the road to responsible sharing of clinical trial data. Clin Trials.

[129] Polanin JR (2018). Efforts to retrieve individual participant data sets for use in a meta-analysis result in moderate data sharing but many data sets remain missing. J Clin Epidemiol.

[130] Polanin JR, Terzian M (2019). A data-sharing agreement helps to increase researchers’ willingness to share primary data: results from a randomized controlled trial. J Clin Epidemiol.

[131] Polanin JR, Williams RT (2016). Overcoming obstacles in obtaining individual participant data for meta-analysis. Res Synth Methods.

[132] Tierney JF, Vale C, Riley R, Smith CT, Stewart L, Clarke M, et al. Individual Participant Data (IPD) Meta-analyses of Randomised Controlled Trials: Guidance on Their Use. PLoS Med. 2015;12(7):e1001855. 10.1371/journal.pmed.1001855. Accessed 15 Apr 2020. 10.1371/journal.pmed.1001855 PMC4510878 26196287

[133] Veroniki AA (2016). Contacting authors to retrieve individual patient data: study protocol for a randomized controlled trial. Trials.

[134] Young T, Hopewell S (2011). Methods for obtaining unpublished data. Cochrane Database Syst Rev.

[135] Lefebvre C, M.E., Glanville J. Chapter 6: Searching for studies In: Higgins JPT, Green S (editors). Cochrane Handbook for Systematic Reviews of Interventions Version 5.1.0 (updated March 2011). The Cochrane Collaboration, 2011; Available from: www.handbook.cochrane.org.

[136] Wolfe N, Gotzsche PC, Bero L (2013). Strategies for obtaining unpublished drug trial data: a qualitative interview study. Syst Rev.

[137] Veroniki AA (2019). Retrieval of individual patient data depended on study characteristics: a randomized controlled trial. J Clin Epidemiol.

[138] Clinical Study Data Request. Our mission. 2019; Available from: https://clinicalstudydatarequest.com/Default.aspx. [cited 2019 April 1].

[139] Murugiah K (2016). Availability of clinical trial data from industry-sponsored cardiovascular trials. J Am Heart Assoc.

[140] NHS Digital (2019). DARS guidance.

[141] NHS Digital (2019). Data Access Request Service (DARS): process.

[142] Clinical Study Data Request (2019). Metrics.

[143] Geifman N (2015). Opening clinical trial data: Are the voluntary data-sharing portals enough?. BMC Med.

[144] Pharmaceutical Research and Manufacturers of America (PhRMA) (2019). Members.

[145] European Federation of Pharmaceutical Industries and Associations (EFPIA) (2019). Membership.

[146] Hopkins AM, Rowland A, Sorich MJ (2018). Data sharing from pharmaceutical industry sponsored clinical studies: Audit of data availability. BMC Med.

[147] Pharmaceutical Research and Manufacturers of America (PhRMA) (2017). EFPIA-PHRMA Princples for responsible clinical trial data sharing report on the 2016 member company survey..

[148] Vivli Center for Global Clinical Research Data (2019). 2018 Progress Report.

[149] Chan AW (2014). Increasing value and reducing waste: addressing inaccessible research. Lancet.

[150] Doshi P, Goodman SN, Ioannidis JP (2013). Raw data from clinical trials: within reach?. Trends Pharmacol Sci.

[151] Gotzsche PC (2011). Why we need easy access to all data from all clinical trials and how to accomplish it. Trials.

[152] Ross JS, Krumholz HM (2013). Ushering in a new era of open science through data sharing: the wall must come down. JAMA.

[153] Fecher B, Friesike S, Hebing M (2015). What drives academic data sharing?. PLoS One.

[154] Nimh Collaborative Data Synthesis for Adolescent Depression Trials Study Team including (2013). Advancing Science Through Collaborative Data Sharing and Synthesis. Perspect Psychol Sci.

[155] Sydes MR, Ashby D (2017). Data authorship as an incentive to data sharing. N Engl J Med.

[156] Bierer BE, Crosas M, Pierce HH (2017). Data authorship as an incentive to data sharing. N Engl J Med.

[157] Berman F, Cerf V (2013). Science priorities. Who will pay for public access to research data?. Science.

[158] Wilhelm EE, Oster E, Shoulson I (2014). Approaches and costs for sharing clinical research data. JAMA.

[159] Zhu CS (2017). Data sharing in clinical trials: an experience with two large cancer screening trials. PLoS Med.

[160] Naudet F (2018). Data sharing and reanalysis of randomized controlled trials in leading biomedical journals with a full data sharing policy: Survey of studies published in the BMJ and PLOS Medicine. BMJ.

[161] Bobrow M (2014). Establishing incentives and changing cultures to support data access.

[162] Rockhold F, Nisen P, Freeman A (2016). Data sharing at a crossroads. N Engl J Med.

[163] Kaiser J (2018). A new portal for patient data. Science.

[164] Tucker K (2016). Protecting patient privacy when sharing patient-level data from clinical trials. BMC Med Res Methodol.

[165] Tudur Smith C (2015). How should individual participant data (IPD) from publicly funded clinical trials be shared?. BMC Med.

[166] European Medicines Agency (2018). External guidance on the implementation of the European Medicines Agency policy on the publication of clinical data for medicinal products for human use.

[167] Mazor KM (2017). Stakeholders’ views on data sharing in multicenter studies. J Comp Eff Res.

[168] van Panhuis WG (2014). A systematic review of barriers to data sharing in public health. BMC Public Health.

[169] Bobrow M (2014). Establishing incentives and changing cultures to support data access.

[170] Bull S, Roberts N, Parker M (2015). Views of ethical best practices in sharing individual-level data from medical and public Health Research: a systematic scoping review. J Empir Res Hum Res Ethics.

[171] Rowhani-Farid A, Allen M, Barnett AG (2017). What incentives increase data sharing in health and medical research? A systematic review. Res Integr Peer Rev.

[172] Kim Y (2017). Fostering scientists’ data sharing behaviors via data repositories, journal supplements, and personal communication methods. Inf Process Manag.

[173] Hopkins C (2016). UK publicly funded clinical trials units supported a controlled access approach to share individual participant data but highlighted concerns. J Clin Epidemiol.

[174] Learned K, Durbin A, Currie R, Beale H, Lam DL, Goldstein T, Salama SR, Haussler D, Morozova O, Bjork I. A critical evaluation of genomic data sharing: Barriers to accessing pediatric cancer genomic datasets: a Treehouse Childhood Cancer Initiative experience [abstract]. In: Proceedings of the American Association for Cancer Research Annual Meeting 2017. Washington, DC. Philadelphia (PA): AACR; Cancer Res. 2017;77(13 Suppl):Abstract nr LB-338. 10.1158/1538-7445.AM2017-LB-338.

[175] Simmonds M, Stewart G, Stewart L (2015). A decade of individual participant data meta-analyses: A review of current practice. Contemp Clin Trials.

[176] Debray TP (2015). Get real in individual participant data (IPD) meta-analysis: a review of the methodology. Res Synth Methods.

[177] Rogozinska E (2017). Meta-analysis using individual participant data from randomised trials: opportunities and limitations created by access to raw data. Evidence-Based Med.

[178] Stewart LA (2015). Preferred reporting items for systematic review and meta-analyses of individual participant data: the PRISMA-IPD statement. JAMA.

[179] SAS Institute (2014). SAS Clinical Trial Data Transparency.

[180] SAS Institute (2018). Multi-Sponsor Environment - SAS® Clinical Trial Data Transparency Version 2.3 User Guide.

[181] Clinical Study Data Request. Access to Data 2018; Available from: https://clinicalstudydatarequest.com/Help/Help-Access-to-Data.aspx. [cited 2018 August 1].

[182] Mospan GA, Wargo KA (2016). Researchers’ experience with clinical data sharing. J Am Board Fam Med.

[183] Mbuagbaw L (2017). Challenges to complete and useful data sharing. Trials.

[184] Aitken M (2016). Public responses to the sharing and linkage of health data for research purposes: a systematic review and thematic synthesis of qualitative studies. BMC Med Ethics.

[185] Hughes S (2014). Preparing individual patient data from clinical trials for sharing: the GlaxoSmithKline approach. Pharm Stat.

[186] Committee on Strategies for Responsible Sharing of Clinical Trial Data, Board on Health Sciences Policy, and Institute of Medicine. Sharing clinical trial data: maximizing benefits, minimizing risk. Washington (DC): National Academies Press (US); 2015 Appendix B, Concepts and Methods for De-identifying Clinical Trial Data]. Available from: https://www.ncbi.nlm.nih.gov/books/NBK285994/. 25590113

[187] Emam KE, Rodgers S, Malin B (2015). Anonymising and sharing individual patient data. BMJ.

[188] Tierney JF (2015). Individual participant data (IPD) meta-analyses of randomised controlled trials: guidance on their use. PLoS Med.

[189] Hollis S (2016). Best practice for analysis of shared clinical trial data. BMC Med Res Methodol.

[190] AllTrials (2014). About AllTrials.

[191] GlaxoSmithKline (2014). Data transparency.

[192] Simmonds MC (2013). Safety and effectiveness of recombinant human bone morphogenetic protein-2 for spinal fusion: a meta-analysis of individual-participant data. Ann Intern Med.

[193] Fu R (2013). Effectiveness and harms of recombinant human bone morphogenetic protein-2 in spine fusion: a systematic review and meta-analysis. Ann Intern Med.

[194] Krumholz HM (2013). A historic moment for open science: the Yale University open data access project and medtronic. Ann Intern Med.

[195] Hrobjartsson A (2013). Why did it take 19 months to retrieve clinical trial data from a non-profit organisation?. BMJ.

[196] Savage CJ, Vickers AJ (2009). Empirical study of data sharing by authors publishing in PLoS journals. PLoS One.

[197] Filippon J (2015). Slow and costly access to anonymised patient data impedes academic research. BMJ.

[198] Tudur Smith C (2014). Sharing individual participant data from clinical trials: an opinion survey regarding the establishment of a central repository. PLoS One.

[199] Huser V, Shmueli-Blumberg D (2018). Data sharing platforms for de-identified data from human clinical trials. Clinical Trials.

[200] So D, Knoppers BM (2017). Ethics approval in applications for open-access clinical trial data: An analysis of researcher statements to clinicalstudydatarequest.com. PLoS One.

[201] Sydes MR (2015). Sharing data from clinical trials: The rationale for a controlled access approach. Trials.

[202] Green AK (2015). The project data sphere initiative: accelerating cancer research by sharing data. Oncologist.

[203] Eichler HG, Sweeney F (2018). The evolution of clinical trials: Can we address the challenges of the future?. Clin Trials.

[204] Khusro A, Aarti C (2017). TB-PACTS: a fresh emphatic data sharing approach. Asian Pac J Trop Dis.

[205] Bertagnolli MM (2017). Advantages of a truly open-access data-sharing model. N Engl J Med.

[206] Asare AL (2016). Clinical trial data access: opening doors with TrialShare. J Allergy Clin Immunol.

[207] Zinner DE, Pham-Kanter G, Campbell EG (2016). The changing nature of scientific sharing and withholding in academic life sciences Research: trends from National Surveys in 2000 and 2013. Acad Med.

[208] Rowhani-Farid A, Barnett AG (2016). Has open data arrived at the British medical journal (BMJ)? An observational study. BMJ Open.

[209] Strom BL (2016). Data sharing - is the juice worth the squeeze?. N Engl J Med.

[210] Bonini S (2014). Transparency and the European medicines agency--sharing of clinical trial data. N Engl J Med.

[211] Drazen JM (2015). Sharing individual patient data from clinical trials. N Engl J Med.

[212] International Consortium of Investigators for Fairness in Trial Data, S (2016). Toward Fairness in Data Sharing. N Engl J Med.

[213] Navar AM, Pencina MJ, Peterson ED (2016). Open access platforms for sharing clinical trial data--reply. JAMA.

[214] Ross JS, Krumholz HM (2016). Open access platforms for sharing clinical trial data. JAMA.

[215] Navar AM (2016). Use of open access platforms for clinical trial data. JAMA.

[216] Garrison NA (2016). A systematic literature review of individuals’ perspectives on broad consent and data sharing in the United States. Genet Med.

[217] Vivli (2019). Aegerion Pharmaceuticals.

[218] Esteve (2014). Commitment letter.

[219] Ipsen (2019). Transparency and trust.

[220] Vivli.org (2020). Vivli Platform Process at a Glance.

