# Supplementary material for: Obtaining and managing data sets for individual participant data meta-analysis: scoping review and practical guide
Source: BMC Med Res Methodol. 2020 May 12;20:113. doi: 10.1186/s12874-020-00964-6 (PMC7218569; doi:10.1186/s12874-020-00964-6)
Supplement: Supplementary file 1 — Additional file 1. [file 12874_2020_964_MOESM1_ESM.docx]

**Appendix – Obtaining and managing data sets for individual participant data meta-analysis: scoping review and practical guide**

## **Appendix A - Search strategy**

Database(s): **Embase** 1974 to 2019 January 04**, OVID Medline Epub Ahead of Print, In-Process & Other Non-Indexed Citations, Ovid MEDLINE(R) Daily and Ovid MEDLINE(R)** 1946 to 2019 January 04

| **#** | **Searches** | **Results** |
| --- | --- | --- |
| 1 | exp clinical trials as Topic/ | 350,553 |
| 2 | "review literature as topic"/ | 40,565 |
| 3 | exp meta-analysis as topic/ | 43,995 |
| 4 | 1 or 2 or 3 | 411,451 |
| 5 | exp information dissemination/ | 24,468 |
| 6 | 4 and 5 | 1,073 |
| 7 | (patient data or participant data).mp. | 30,123 |
| 8 | 6 and 7 | 35 |
| 9 | 4 and 7 | 2,444 |
| 10 | limit 9 to (clinical trial, all or meta analysis) [Limit not valid in Embase; records were retained] | 1,113 |
| 11 | 9 not 10 | 1,331 |
| 12 | 5 and 7 | 124 |
| 13 | research subjects/ | 7,195 |
| 14 | 4 and 13 | 1,133 |
| 15 | limit 14 to (clinical trial, all or meta analysis) [Limit not valid in Embase; records were retained] | 42 |
| 16 | 13 not 14 | 6,062 |
| 17 | access to information/ and 7 | 170 |
| 18 | 8 or 9 or 12 or 14 or 15 or 17 | 3,797 |
| 19 | data transparency.mp. [mp=ti, ab, hw, tn, ot, dm, mf, dv, kw, fx, dq, nm, kf, px, rx, an, ui, sy] | 231 |
| 20 | data sharing.mp. [mp=ti, ab, hw, tn, ot, dm, mf, dv, kw, fx, dq, nm, kf, px, rx, an, ui, sy] | 6,011 |
| 21 | 19 or 20 | 6,211 |
| 22 | 1 and 21 | 389 |
| 23 | 18 or 22 | 4,130 |
| 24 | remove duplicates from 23 | 3,433 |

## **Appendix B - Identifying clinical trials using the International Clinical Trials Registry Platform**

The goal of the World Health Organization’s International Clinical Trials Registry Platform (ICTRP) is to make information about all clinical trials involving humans publicly available to all parties involved in health care decision making [1]. The ICTRP groups clinical trial registration data regarding the same study from 17 primary registries which adhere to the ICTRP standards. Registries accessed through the ICTRP include clinicaltrials.gov, the European Union Clinical Trials register (EU-CTR) and several other clinical trial registries from around the world [1]. The ICTRP standards focus on the content, quality and validity, accessibility, unambiguous identification, technical capacity, administration and governance of a clinical trial registry. Trial registration and enforcement remains a problem in many countries, however practices are generally improving [2].

Users can access the ICTRP search engine (<https://apps.who.int/trialsearch/>) to identify clinical trials relevant to their systematic review and meta-analysis. The ICTRP search engine is free to use, regularly updated, and available in six languages: English, French, Spanish, Arabic, Chinese and Russian.

Clinical trials can be searched by their title, condition, intervention, study sponsor, country of recruitment, date of registration or recruitment status. Clicking on any clinical trial will display the “Trial Registration Data Set” (TRDS), which is a list of 20 items a trial should supply for registration [3]. The trial title, contact information of primary investigators, and interventions tested are among the useful items in the TRDS. Users are able to export this data which can assist in maintaining organization of important trial data, contact information, and recent events throughout data acquisition and analysis.

TRDS items listed in the ICTRP may be incomplete or lacking sufficient detail, especially for industry sponsored trials [5-7]. Of trials registered during the year 2012, Viergever and Ghersi (2014) report that approximately 15% did not include contact name, approximately 30% did not provide a telephone number and approximately 33% did not include an email address [6]. Differences in formatting, quality control and enforcement procedures between trial registries accessed by the ICTRP are thought to be the cause. These statistics, as well as timely trial registration practices, have improved compared to previous years, and are expected to continue improvement as registries evolve alongside political legislation and industry standards [5, 6].

When the contact information or any other TRDS field is improperly reported, the IPDMA study coordinator may be able to access further details from the original trial registration webpage through a link provided in the TRDS [8]. Further contact information and other important study details can be found in the study protocols or results manuscripts, provided they have been published. Outstanding details may require the study coordinator to search the internet for answers in faculty registries, sponsor websites, other trial specific websites, or through personal contact with known trial contributors.

## **Appendix C - Government Agencies such as the US Food and Drug Administration (FDA) and European Medicines Association (EMA)**

If the intervention of interest has sought regulatory approval, researchers may consider submitting a Freedom of Information Act (FOIA) request through federal agencies such as the U.S. Department of Health & Human Services (HHS) or the EMA. Beginning January 1, 2015, the clinical study reports for medicines which have received marketing authorisation are published by the EMA. The information is not to be used for commercial purposes but is available for the public to browse on-screen, download and print. Information considered to be commercially confidential will be redacted [9]. Presently, obtaining individual participant data from either the FDA or EMA is unlikely. However, the EMA is consulting with patients, healthcare workers, researchers and the pharmaceutical industry to incorporate the publication of individual participant data into future versions of their recent policy on the sharing of clinical data [9]. The FDA is also in the process of developing policies granting access to clinical trial data, yet no formal deadlines are in place [10].

Contact information, a description of available data provided by the FDA as well as the webpage to submit a FOIA request is found at the following links; <https://www.fda.gov/RegulatoryInformation/FOI/default.htm> [11] and <https://wcdapps.hhs.gov/FoiaRequest/> [12]. Requested (aggregate) data can often be provided in as little as one month but may also take longer depending on availability of the requested information.

The process for submitting a request or searching for data is relatively straightforward and fast. The most integral portion of the request is clearly communicating the exact information of interest. Due to the large amount of available data, it is important that requesters clearly identify the socio-demographic, exposure and outcome variables of interest. When discussing the interventions of interest, specific identifiers such as the New Drug Application (NDA) or National Drug Code (NDC) numbers, in addition to the product name should be noted. These numbers can be found by searching the FDA Drug Approvals and Database, <http://www.fda.gov/Drugs/InformationOnDrugs/default.htm> [13]. Requests without this information may be denied, requiring the investigator to resubmit a request with more explicit details, adding to the amount of effort and time required to obtain study information. Costs may be incurred but are often relieved if the project is of scientific nature, without financial motivation, and is beneficial to public health. Contacting the EMA, FDA, or other appropriate federal agency prior to submitting a request will help to clarify these issues in advance.

## **Appendix D - Example email text to request individual patient data from principal investigators or sponsors of trials**

Hello <STATE NAME>,

My name is <STATE NAME>, I am <STATE TITLE AND AFFILIATION> emailing you on behalf of the <STATE RESEARCH TEAM NAME>, led by <STATE NAME>. We are conducting an individual participant data meta-analysis investigating (IPDMA) <STATE PURPOSE OF IPDMA>. The clinical trial <STATE TRIAL NAME; REGISTRATION NUMBER>, which <STATE ROLE OF EMAIL RECIPIENT>, has met our inclusion criteria.

With your permission, we would like to incorporate individual participant data from <STATE TRIAL> in our analysis. In exchange for sharing <TRIAL NAME> data we are pleased to offer you authorship on all subsequent results publications. I have attached our study protocol to this email for further clarification of our study objectives and analysis plans.

The <STATE TRIAL> is an important addition to our IPDMA. If you should have any questions regarding the project or your role within it, please do not hesitate to ask. We would be happy to provide any further documentation or discuss the next steps of this project via email or teleconference.

Thank you very much for considering our request, it is greatly appreciated.

<STATE NAME>

<STATE AFFILIATION>

## **Appendix E - Example of a data sharing agreement**

**DATE:**

**INSERT LOGO DATA REQUESTING ORGANIZATION**

(1) **<DATA SHARING ORGANIZATION>**

(2)  **<DATA REQUESTING ORGANIZATION>**

**Data Transfer Agreement**

**THIS AGREEMENT** dated **<INSERT DATE>** is made **BETWEEN:**

(1) **<DATA SHARING ORGANIZATION>** whose administrative offices are located at **<STATE ADDRESS>**

(2) **<DATA REQUESTING ORGANIZATION>** whose registered office/principal place of business is at **<STATE ADDRESS>**

**WHEREAS:**

(A) <DATA SHARING ORGANIZATION> lead a project known as <**TRIAL TITLE AND REGISTRATION NUMBER**> which has generated a data set (the “Data”); and

(B) The Data is under custodianship of <DATA SHARING ORGANIZATION> ; and

(C) <**DATA REQUESTING ORGANIZATION**> is undertaking a research project as further described in Schedule 2 (the “Programme of Work”); and

(D) <DATA SHARING ORGANIZATION> is willing to provide a copy of the Data to <**DATA REQUESTING ORGANIZATION**> for use in the Programme of Work subject to the following terms and conditions.

NOW IT IS AGREED as follows:

1. **GRANT OF LICENCE**
   1. The Data shall remain at all times the property of <DATA SHARING ORGANIZATION>.
   2. To the extent that <DATA SHARING ORGANIZATION> is permitted to grant such rights, and subject to clause 3.1, <DATA SHARING ORGANIZATION> grants to <**DATA REQUESTING ORGANIZATION**> a non-exclusive, non-transferable licence to use the Data for non‑commercial research as specifically described in the Programme of Work detailed in Schedule 2 for the Term specified in Schedule 4.
   3. <**DATA REQUESTING ORGANIZATION**> is permitted to make additional copies of the Data solely for use as described in the Programme of Work by <**DATA REQUESTING ORGANIZATION**> researchers.
   4. <**DATA REQUESTING ORGANIZATION**> will not transfer the Data to any other body, or permit their use within <**DATA REQUESTING ORGANIZATION**> other than by the research group specified in Schedule 2, without (in each case) prior written consent from <**DATA SHARING ORGANIZATION**>.
   5. The Data will be used only for the Programme of Work and will not be used to carry out any other studies without prior written consent from <**DATA SHARING ORGANIZATION**>.
   6. <**DATA REQUESTING ORGANIZATION**> shall take appropriate measures to ensure the security of such Data and guard against unauthorised access thereto or disclosure thereof or loss or destruction while in its custody.
   7. <**DATA SHARING ORGANIZATION**> shall take appropriate measures to ensure the Data is anonymised before transfer to **<DATA REQUESTING ORGANIZATION>** and that the key to personal identities to all persons to whom the Data relates is kept in a separate and secure place.
2. **PAYMENT**
   1. The data are provided fee of charge. No payment is required. However, as consideration for the data provided by <**DATA SHARING ORGANIZATION**>, <**DATA REQUESTING ORGANIZATION**> will
      1. fulfill information obligation according to this contract (e.g. 5.3.) towards <**DATA SHARING ORGANIZATION**>,
      2. allow <**DATA SHARING ORGANIZATION**> a non-exclusive right to use the data and results generated in the Programme of Work, for scientific use such as for example scientific information for healthcare professionals, internal research planning, and scientific discussions with healthcare professionals; this right shall also be transferable to affiliated companies of <**DATA SHARING ORGANIZATION**>. For an intended *commercial* use, section 5.4 shall apply.
      3. <**DATA SHARING ORGANIZATION**> may have access to study results and publically available data only once results have been published. <**DATA SHARING ORGANIZATION**> may not have access to individual patient data provided by other research groups or as far as Data sharing agreements with other research groups preclude **<DATA REQUESTING ORGANIZATION>** from sharing their data with third parties.
3. **WARRANTIES AND INDEMNITIES**
   1. <**DATA REQUESTING ORGANIZATION**> understands that <**DATA SHARING ORGANIZATION**> makes no representations and gives no warranties of any kind in relation to the Data. In particular, no warranties are given about quality or fitness for a particular purpose; or that the use of the Data will not infringe any intellectual property or other rights; or that the Data do not contain any defamatory material.
   2. <**DATA SHARING ORGANIZATION**> will not be liable for any use made of the Data by **<DATA REQUESTING ORGANIZATION>** including without limitation publication under clause 4.7.
4. **CONFIDENTIALITY AND PUBLICATION PROCEDURES**
   1. For the purpose of this clause, “Confidential Information” means all and any specifications, drawings, circuit diagrams, tapes, discs and other computer-readable media, documents, information, techniques and know-how, including but not limited to the Data, which are disclosed by one Party to the other in connection with the Programme of Work and marked or labelled “Proprietary”, “Confidential” or “Sensitive” by the disclosing party at the time of disclosure; or are written, prepared or generated in the course of, and as part of, the Programme of Work. For clarify, Confidential Information excludes the final results of any analysis created or produced using the Confidential Information where such results were created or produced in accordance with the Programme of Work under this Agreement.
   2. Subject to clauses 4.3 and 4.5, each party will use its reasonable endeavours not to disclose to any third party any Confidential Information received from the other party.
   3. Clause 4.2 will not apply to any Confidential Information which:
      1. is known to the receiving party before disclosure, and not subject to any obligation of confidentiality owed to the disclosing party; or
      2. is or becomes publicly known without the fault of the receiving party; or
      3. is obtained by the receiving party from a third party in circumstances where the receiving party has no reason to believe that there has been a breach of an obligation of confidentiality owed to the disclosing party; or
      4. the receiving party can establish by reasonable proof was substantially and independently developed by officers or employees of the receiving party who had no knowledge of the disclosing party’s Confidential Information; or
      5. is approved for release in writing by an authorised representative of the disclosing party; or
      6. the receiving party is required to disclose by law or regulation (provided that, in the case of the Freedom of Information Act 2000, none of the exceptions to that Act applies to the information disclosed) or by order of a competent authority (including any regulatory or governmental body or securities exchange); provided that, where practicable, the disclosing party is given reasonable advance notice of the intended disclosure and provided that the relaxation of the obligation of confidentiality shall only last for as long as necessary to comply with the relevant law, regulation or order and shall apply solely for the purposes of such compliance.
   4. If any party to this Agreement receives a request under the Freedom of Information Act 2000 to disclose any information that, under this Agreement, is Confidential Information, it will notify and consult with the other party(ies) as applicable. The other party(ies) will respond within 5 days after receiving notice if that notice requests the other party(ies) to provide information to assist in determining whether or not an exemption to the Freedom of Information Act 2000 applies to the information requested under that Act.
   5. Subject to clause 4.7 below, each party’s duty to protect Confidential Information received under this Agreement shall survive the termination of the Programme of Work and continue in full force and effect for the period of five (5) years thereafter.
   6. The Parties acknowledge that all personal information and personal health information provided to it hereunder is subject to applicable privacy legislation that may include Canada’s *Personal Information Protection and Electronic Documents Act* (PIPEDA) and the Data Protection Act, 1998, The Parties warrant that it and/or its designee will adhere to and comply with applicable laws and regulations regarding protection of personal information and personal health information.

- 1. Where **<DATA REQUESTING ORGANIZATION>** wishes to submit for publication results of the Programme of Work in which <**DATA SHARING ORGANIZATION**> have provided the Data pursuant to this Agreement, **<DATA REQUESTING ORGANIZATION>** will submit a copy of the publication to <**DATA SHARING ORGANIZATION**> in writing not less than fifteen (15) days in advance of the submission for publication for review. **<DATA REQUESTING ORGANIZATION>** agrees to take into consideration any reasonable comments made by <**DATA SHARING ORGANIZATION**> regarding the proposed publication with respect to the Data and shall not unreasonably refuse to include amendments requested by <**DATA SHARING ORGANIZATION**> where these relate to the Data. Notwithstanding the foregoing, the final analysis and interpretation of the Data by **<DATA REQUESTING ORGANIZATION>** in the performance of the Programme of Work shall remain with **<DATA REQUESTING ORGANIZATION>**. <**DATA SHARING ORGANIZATION**> shall notify **<DATA REQUESTING ORGANIZATION>** in writing with its comments regarding the proposed publication within fifteen (15) days after the receipt of the publication by <**DATA SHARING ORGANIZATION**>, failing which **<DATA REQUESTING ORGANIZATION>** shall be free to assume that <**DATA SHARING ORGANIZATION**> has no objection to the proposed publication.
  2. Authorship of publications in clause 4.8 shall be determined in accordance with ICMJE guidelines.

1. **INTELLECTUAL PROPERTY RIGHTS**
   1. This Agreement does not affect the ownership of any Intellectual Property in the Data and the Intellectual Property in them will remain the property of <**DATA SHARING ORGANIZATION**>. No licence to use any Intellectual Property is granted or implied by this Agreement except the rights expressly granted in this Agreement.
   2. Nothing in this agreement shall restrict <**DATA SHARING ORGANIZATION**> right to licence the Data to other commercial and non-commercial entities.
   3. **<DATA REQUESTING ORGANIZATION>** shall promptly inform <**DATA SHARING ORGANIZATION**> of any information arising from the Programme of Work, including but not limited to, information combined or incorporated with the Data, including any Intellectual Property, whether patentable or not, which are capable of exploitation either by direct adoption into the healthcare service or via commercialisation. Without the written consent of <**DATA SHARING ORGANIZATION**>, **<DATA REQUESTING ORGANIZATION>** shall not, alone, or together with one or more others, file any patent applications on inventions arising from/made by use of the Data.
   4. If **<DATA REQUESTING ORGANIZATION>** or <**DATA SHARING ORGANIZATION**> wishes to use the information arising from the Programme of Work for clinical patient care or for commercial use each party agrees to negotiate in good faith for the grant of an appropriate licence or conclusion of a revenue sharing agreement, if justified, taking into consideration third party funder and regulatory requirements.
2. **ASSIGNMENT**
   1. This Agreement is not transferable, nor any of the rights granted in it (with the exception of 2.1.2), and no party may purport to assign it (in whole or in part) without the prior written consent of the other.
3. **TERM AND TERMINATION**
   1. This Agreement shall have effect for the Term stated in Schedule 4 unless terminated earlier in accordance with the provisions set out below.
   2. Any party may at any time terminate this Agreement, by written notice to the others. Upon termination, **<DATA REQUESTING ORGANIZATION>** and any party who may have received Data under clause 1.4 shall immediately cease all use of the Data received pursuant to this Agreement; and within eight (8) days thereafter shall destroy or return to <**DATA SHARING ORGANIZATION**> all copies of Data and related information in its possession, sending written confirmation of despatch or destruction to Novartis.
4. **LIMITATION OF LIABILITY**
   1. <**DATA SHARING ORGANIZATION**> makes no representation or warranty that advice or information given by its employees, students, agents or appointees, or the content or use of any materials, works or information provided in connection with the Data, will not constitute or result in infringement of third-party rights.
   2. <**DATA SHARING ORGANIZATION**> accepts no responsibility or liability for any use which may be made of the Data or **<DATA REQUESTING ORGANIZATION>**’s use of the results of the Programme of Work, nor for any reliance which may be placed on the Data, nor for advice or information given in connection with them.
   3. **<DATA REQUESTING ORGANIZATION>** undertakes to make no claim in connection with this Agreement or its subject matter against any employee, student, agent or appointee of <**DATA SHARING ORGANIZATION**> (apart from claims based on fraud or wilful misconduct). This undertaking is intended to give protection to individual researchers: it does not prejudice any right which **<DATA REQUESTING ORGANIZATION>** might have to claim against <**DATA SHARING ORGANIZATION**>. The benefit conferred by this sub-clause is intended to be enforceable by the persons referred to in it.
   4. Subject to clause 8.6, the liability of any party to the other for any breach of this Agreement, for any negligence, or arising in any other way out of the subject-matter of this Agreement, the Programme of Work or the results will not extend to any indirect damages or losses, or to any loss of profits, loss of revenue, loss of business, loss of data, loss of contracts or opportunity, whether direct or indirect, even if, in any such case, the party bringing the claim has advised the other of the possibility of those losses or if they were within the other party’s contemplation.
   5. Nothing in this Agreement limits or excludes either party’s liability for:
      1. death or personal injury resulting from negligence; or
      2. any fraud or for any sort of other liability which, by law, cannot be limited or excluded.
   6. If any sub-clause of this clause 8 is held to be invalid or unenforceable under any applicable statute or rule of law then it shall be deemed to be omitted, and if as a result any party becomes liable for loss or damage which would otherwise have been excluded then such liability shall be subject to the remaining sub-clauses of this clause 8.
5. **GENERAL**
   1. This Agreement shall be interpreted in accordance with the Laws of <**STATE COUNTRY**>. The competent court shall be <**STATE LOCATION**>.
   2. Clause headings are inserted in this Agreement for convenience only, and they shall not be taken into account in the interpretation of this agreement.
   3. Nothing in this Agreement shall create, imply or evidence any partnership or joint venture between <**DATA SHARING ORGANIZATION**> and the Licensee or the relationship between them of principal and agent.
   4. Any variation of this Agreement shall be in writing and signed by authorised representatives for both parties.
   5. **<DATA REQUESTING ORGANIZATION>** shall not assign, subcontract or transfer any rights, duties or obligations under this agreement without the prior written approval of <**DATA SHARING ORGANIZATION**>.

**SCHEDULES**

1. Description of the Data

2. Programme of Work

3. Payment

4. Term

**AS WITNESS** the hands of authorised signatories for the parties on the date first mentioned above.

**SIGNED** for and on behalf of **<DATA SHARING ORGANIZATION>**:

Name:

Position: Corresponding author, <TRIAL TITLE>

Signed:

**SIGNED** for and on behalf of **<DATA REQUESTING ORGANIZATION>** industry liason office

Name:

Position:

Signature:

Read and Acknowledged by **<DATA REQUESTING ORGANIZATION>** primary investigator

Name:

Position:

Signature:

**Schedule 1**

**The Data**

**Includes:**

Data from the clinical trial entitled <STATE NAME OF CLINICAL TRIAL AND REGISTRATION NUMBER>

**Excludes:**

LIST ANY EXCLUSIONS

**Schedule 2**

**Programme of Work**

**PI and Name of Institution’s Research Group**

<**INSERT STUDY PROTOCOL**>

The **Programme of Work** does not extend to:

- <**DATA REQUESTING ORGANIZATION**> commercially exploiting the Data;
- nor to the evaluation or any other use of the Data by <**DATA REQUESTING ORGANIZATION>** outside the Programme of Work described above.

**Schedule 3**

**Payment**

**<DATA REQUESTING ORGANIZATION>** shall pay to <**TRIAL SPONSOR**> The sum of $0 plus VAT.

**Schedule 4**

**Term**

This Agreement shall have an Effective Date of last date of signature and shall terminate 1 year later (the “Term”)

**References Appendix**

1. World Health Organization. *International Clinical Trials Registry Platform (ICTRP),*. 2014; Available from: <http://www.who.int/ictrp/en/>.

2. Ghersi, D. and T. Pang, *From Mexico to Mali: four years in the history of clinical trial registration.* J Evid Based Med, 2009. **2**(1): p. 1-7.

3. *International Clinical Trials Registry Platform (ICTRP): Registry Network*. 2014 [cited 2014 May 12]; Available from: <http://www.who.int/ictrp/network/trds/en/>.

4. *Microsoft Excel, Part of Microsoft Office Professional Plus 2010,*, in *Microsoft Office Professional Plus 2010*. 2010, Microsoft Corporation.

5. Viergever, R.F. and D. Ghersi, *The quality of registration of clinical trials.* PLoS One, 2011. **6**(2): p. e14701.

6. Viergever, R.F., et al., *The quality of registration of clinical trials: still a problem.* PLoS One, 2014. **9**(1): p. e84727.

7. Wieseler, B., et al., *Impact of document type on reporting quality of clinical drug trials: a comparison of registry reports, clinical study reports, and journal publications.* BMJ, 2012. **344**: p. d8141.

8. World Health Organization. *International standards for clinical trial registries*. 2012; Available from: <http://apps.who.int/iris/bitstream/10665/76705/1/9789241504294_eng.pdf?ua=1>.

9. European Medicines Agency. *European Medicines Agency policy on publication of clinical data for medicinal products for human use*. 2014 [cited 2014 October 3]; Available from: <http://www.ema.europa.eu/docs/en_GB/document_library/Other/2014/10/WC500174796.pdf>.

10. Food and Drug Administration (FDA). *Availability of Masked and De-identified Non-Summary Safety and Efficacy Data; Request for Comments*. 2013 [cited 2014 June 20]; Available from: <https://federalregister.gov/a/2013-13083>.

11. U.S. Food and Drug Administration (FDA). *Freedom of Information*. [cited 2014 July 1]; Available from: <http://www.fda.gov/RegulatoryInformation/FOI/default.htm>.

12. U.S Department of Health and Human Services (HHS). *Submit a FOIA request*. [cited 2014 June 22]; Available from: <http://wcdapps.hhs.gov/FoiaRequest/>.

13. U.S. Food and Drug Administration (FDA). *Drug Approvals and Databases*. 2014 [cited 2014 July 1]; Available from: <http://www.fda.gov/Drugs/InformationOnDrugs/default.htm>.
